# Multimorbidity and analgesic-related harms: a systematic review

**DOI:** 10.1016/j.bja.2025.02.012

**Published:** 2025-03-20

**Authors:** Christopher H. Grant, Heather Walker, Karen N. Barnett, Patrick B. Mark, Lesley A. Colvin, Samira Bell

**Affiliations:** 1 Division of Population Health and Genomics, School of Medicine, University of Dundee, Dundee, UK; 2 School of Cardiovascular and Metabolic Health, College of Medical, Veterinary and Life Sciences, University of Glasgow, Glasgow, UK

**Keywords:** analgesics, drug-related harms, multiple long-term conditions, multimorbidity, opioids, serious adverse effects

## Abstract

**Background:**

Multimorbidity is the presence of two or more long-term medical conditions. Chronic pain affects more than half of people with multimorbidity, and optimal treatment strategies are unknown. We aimed to quantify the risk of adverse outcomes from the following analgesics: opioids, nonsteroidal anti-inflammatory drugs (NSAIDs), and gabapentinoids in adults with multimorbidity.

**Method:**

The review was registered on PROSPERO (CRD42023462592). We searched Medline, CINAHL, Web of Science, Embase, and CENTRAL for studies reporting analgesic-related harms in people with multimorbidity or the impact of multimorbidity on harms in adults exposed to analgesics. Two researchers independently screened titles/abstracts, completed full-text reviews, extracted data, and assessed risk of bias using the Newcastle-Ottawa scale. Studies were synthesised narratively, grouping by analgesic class and direction of effect.

**Results:**

We screened 6690 records and 344 full texts, with 27 studies included (*n*=2 671 958 patients). Studies were heterogenous, with variable quality (high risk of bias, *n*=11). Most studies on opioids reported adverse outcomes (12/16). Opioid use compared with non-use was associated with increased mortality in adults with multimorbidity. Multimorbidity was associated with opioid overdose and death among adults prescribed opioids for pain. Half of studies of NSAIDs reported adverse outcomes (6/11) including gastrointestinal bleeding. Only one study assessed gabapentinoids which found an association with delirium and pneumonia, but not mortality in people with multimorbidity.

**Conclusions:**

There is evidence of harms associated with opioids in adults with multimorbidity, including overdose and increased mortality. There is a lack of evidence on gabapentinoids. Further research is required to understand optimal analgesic management in people with multimorbidity.

**Systematic review protocol:**

PROSPERO (CRD42023462592).

**Editor's key points**

• Chronic pain affects more than half of people with multimorbidity (more than one long-term condition). Optimal analgesic strategies are unknown. Such patients are under-represented in trials.
• This review article summarises the available evidence on analgesic-related harms in adults with multimorbidity. There is evidence of harm associated with opioid use, including overdose and mortality, and an absence of evidence related to gabapentinoids.
• Caution should be exercised when prescribing opioids to multimorbid patients. Further research is needed to quantify the benefits and harms of analgesics, specifically NSAIDs and gabapentinoids.

Multimorbidity is the presence of two or more long-term physical or mental health conditions^1^^,^^2^ and affects more than a third of adults worldwide.^3^ Multimorbidity increases with age and socioeconomic deprivation and is associated with reduced quality of life and premature mortality.^1^^,^^4^, ^5^, ^6^, ^7^ Furthermore, multimorbidity represents a major challenge for global health care systems, which are orientated towards disease-specific management.^8^^,^^9^

Chronic pain affects more than half of people with multimorbidity^10^^,^^11^ with limited management options. Guidelines for people with multimorbidity do not make recommendations on the treatment of pain,^1^^,^^12^^,^^13^ nor do guidelines on chronic pain management make specific recommendations for people with multimorbidity.^14^, ^15^, ^16^ The prevalence of analgesic use in those with multiple long-term conditions (LTCs) varies by setting (e.g. primary or secondary care) and has been reported to be as many as three in four hospital inpatients.^17^

People with multimorbidity are under-represented in clinical trials^18^ and so the safety of analgesic use among this population has not been established. Pharmacological treatments for pain are often associated with adverse events. Opioids, gabapentinoids, and nonsteroidal anti-inflammatory drugs (NSAIDs) are three of the most widely prescribed analgesics.^19^, ^20^, ^21^, ^22^, ^23^, ^24^ Opioids can lead to small reductions in pain intensity, but are also associated with increased risk of overdose, abuse, fractures, and myocardial infarction,^25^^,^^26^ with little long-term data on the safety of opioids for chronic non-cancer pain despite widespread use.^25^, ^26^, ^27^, ^28^ Gabapentinoids have neurological sequelae (e.g. sedation and ataxia) in addition to abuse potential^29^, ^30^, ^31^, ^32^, ^33^, ^34^ and possible increased mortality.^35^ NSAIDs are associated with gastrointestinal complications, acute kidney injury (AKI), and cardiovascular events.^36^, ^37^, ^38^, ^39^, ^40^ A meta-analysis reporting opioid outcomes found that observational and interventional studies of older adults (>65 yr) excluded those with substantial comorbidities.^41^^,^^42^ Although some studies have assessed the impact of comorbid conditions in isolation, there is a lack of studies examining the adverse effects of analgesics in people with multimorbidity. It is likely that the lack of consistency regarding the measurement of multimorbidity has hindered appropriate study^43^, ^44^, ^45^ with recent consensus recommending that multimorbidity be measured by LTC count or weighted comorbidity scoring index, depending on the study purpose.^46^ The harms of analgesics by such measures have received little attention despite most opioid-related hospitalisations occurring in adults with multimorbidity.^47^

The aim of this systematic review was to quantify the risk of adverse outcomes from opioids, NSAIDs, and gabapentinoids in adults with multimorbidity.

## Methods

This systematic review was registered prospectively on PROSPERO (registration number: CRD42023462592). The results are reported with reference to the Preferred Reporting Items for Systematic Reviews and Meta-Analyses (PRISMA) 2020 statement.^48^

### Eligibility criteria

To be eligible for inclusion, studies had to record and report multimorbidity using a recommended measure (i.e. a weighted comorbidity scoring index or LTC count)^46^ in adults (aged 18 yr or older). Studies involving only people with multimorbidity or where the population was reported to have a mean/median of two or more LTCs and who were exposed to opioids, NSAIDs (non-aspirin) or gabapentinoids for pain, and followed for drug-related harms, were eligible. Studies of general populations that did not report baseline multimorbidity were only eligible if the independent association of a recommended measure of multimorbidity^46^ on adverse events was reported among adults prescribed relevant analgesics for pain. An adverse event related to analgesic use was defined according to the PRISMA harms checklist as ‘*an unfavourable outcome that occurs during or after the use of a drug’ for which ‘the causal relation between the intervention and the event is at least a reasonable possibility*’.^49^, ^50^, ^51^ Drug-related harms therefore included any harm reported regardless of whether it was considered serious, that could be potentially related to the consumption of a relevant analgesic. Empirical quantitative studies, including observational and interventional (i.e. whereby investigators actively intervened as part of the study design^52^), were eligible for inclusion. Studies that did not report a recognised measure of multimorbidity or drug-related harms were excluded. Studies were also excluded if the multimorbidity measure and the relevant analgesic were treated as covariates in a multivariable model such that the relationship of interest could not be isolated. Non-English language, qualitative and non-primary reports (e.g. narrative review articles) were ineligible. Full details of eligibility criteria are listed in Supplementary material S1.

### Search strategy and study selection

The search strategy was devised with the assistance of an academic librarian (SM), using a combination of index terms and keywords, with reference to Cochrane guidance on identifying studies of adverse effects.^53^ Medline (PubMed), CINAHL (Ebsco), Web of Science, Embase (Ovid), and CENTRAL (Cochrane Library) were searched from inception to September 30, 2024. Records were imported into Covidence software for study selection.^54^ Titles/abstracts were screened and potentially relevant texts sourced for full eligibility assessment by two independent researchers (CHG/HW). Disagreements were resolved by consensus, a third reviewer (SB), or both. The search strategy for all databases is provided in Supplementary Table S1.

### Data extraction and risk of bias

A data extraction form was created to record study characteristics (e.g. design, setting, sample size, dates), participants (e.g. inclusion/exclusion criteria, multimorbidity definition/measure, patient characteristics, comorbidities), analgesic exposure (e.g. medication, form, dose, data sources), adverse outcomes (e.g. definition/measure, timing, missing data, loss to follow-up), and statistical method (e.g. analysis, adjustments, censoring).

The Newcastle-Ottawa Scale^55^ was used to assess risk of bias in observational studies covering selection, comparability, and outcome/exposure domains with required modifications (Supplementary material S2). It has been used in previous reviews of analgesic-related harms^56^^,^^57^ and multimorbidity.^3^ The risk of bias in non-randomised studies of interventions (ROBINS-I)^58^ tool was used to assess bias in interventional studies.

Data from each study were extracted and studies assessed for quality by two independent reviewers (CHG/HW) with discrepancies resolved by consensus, a third reviewer (SB), or both.

### Data synthesis

It was expected that statistical synthesis through meta-analyses would not be possible because of substantial methodological and contextual heterogeneity. Studies were therefore grouped into analgesic class for the purposes of synthesis to generate clinically relevant results. Studies were further subdivided based on direction of association/effect (i.e. evidence of harm associated with multimorbidity or analgesic exposure *vs* no evidence of harm) and described according to setting (i.e. secondary care, primary care or both) because of inconsistencies in the effect measures and data reported across studies.^59^ The numerical results included in the synthesis are displayed in tabular form consistent with in-text description.

## Results

### Study selection

**Fig 1:**
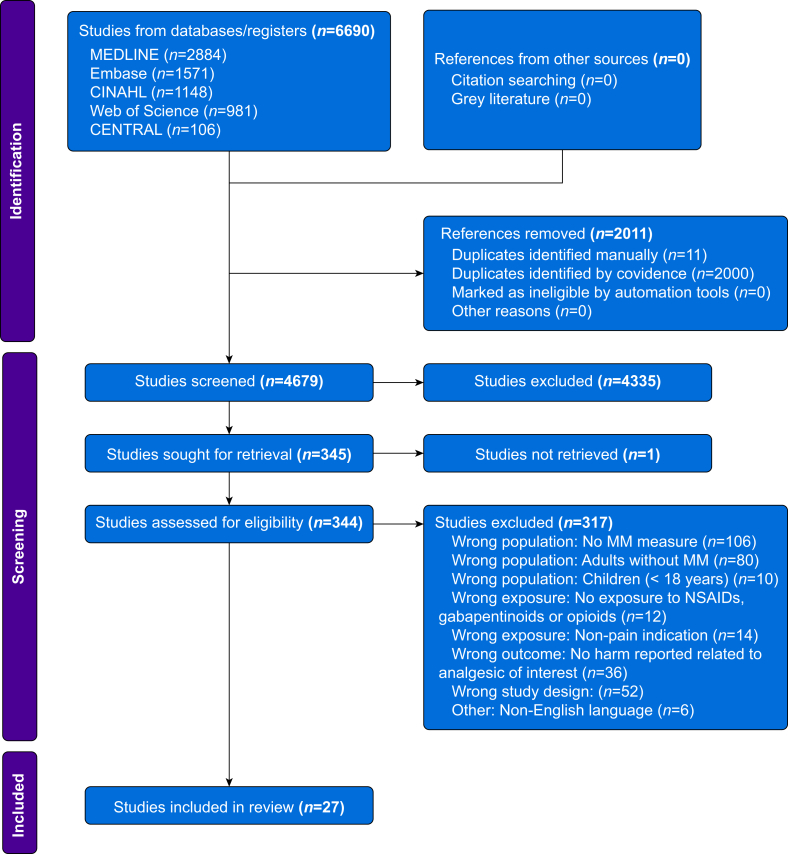
PRISMA flow diagram. MM, multimorbidity; PRISMA, Preferred Reporting Items for Systematic Reviews and Meta-Analyses.

A systematic search of the literature returned 6690 records. After the removal of duplicates, 4679 titles/abstracts were screened and 344 full texts retrieved for eligibility assessment. A total of 27 studies^60^, ^61^, ^62^, ^63^, ^64^, ^65^, ^66^, ^67^, ^68^, ^69^, ^70^, ^71^, ^72^, ^73^, ^74^, ^75^, ^76^, ^77^, ^78^, ^79^, ^80^, ^81^, ^82^, ^83^, ^84^, ^85^, ^86^ were included in the review. Reasons for exclusions are detailed in Figure 1.

### Characteristics of included studies

Twenty seven studies included data from 2 671 958 people. A diverse range of methodologies was observed with substantial variability in study design including: setting, population(s), multimorbidity measures, quantification of analgesics, comparisons, and outcomes. Sixteen studies assessed opioids,^60^, ^61^, ^62^, ^63^, ^64^, ^65^, ^66^, ^67^, ^68^, ^69^, ^70^, ^71^, ^72^, ^73^, ^74^^,^^86^ 11 examined NSAIDs,^74^, ^75^, ^76^, ^77^, ^78^, ^79^, ^80^, ^81^, ^82^, ^83^^,^^85^ and one study focused on gabapentinoids.^84^ One study reported results for both opioids and NSAIDs, separately.^74^ Basic descriptors of included studies are summarised in Table 1.

**Table 1:** Basic descriptors of included studies, *n*=27. ACG, adjusted clinical group; ADG, aggregated diagnosis group; AKI, acute kidney injury; CCI, Charlson Comorbidity Index; CV, cardiovascular; ECI, Elixhauser comorbidity index; ED, emergency department; GI, gastrointestinal; HAM-D 17, Hamilton Depression Rating Scale; HbA1C, haemoglobin A1c; HIV, human immunodeficiency virus; LOS, length of stay; LTC, long-term condition; MM, multimorbidity; MMSE, mini-mental state exam; NSAID, nonsteroidal anti-inflammatory drugs; N/R, not reported; OA, osteoarthritis; ORADE, opioid-related adverse drug event; rDANCAMI score, Danish Comorbidity Index for Acute Myocardial Infarction restricted to non-cardiovascular diseases; SF-12, Short Form-12 survey; TMA, thrombotic microangiopathy. ∗Individually reported.

Reference	Country	Study design	Study dates	Setting	Population	MM measure	Analgesic(s)
Podesser and colleagues, 2024^86^	Austria	Case control	June 2016–March 2018	Secondary	Older (≥65 yr) adult inpatients with dementia from a single-centre geronto-psychiatric hospital	LTC count	Opioids
Cano-Escalera and colleagues, 2023^60^	Spain	Prospective cohort	Sep 2017–Jan 2021	Secondary	Hospital adult inpatients (internal medicine or neurology)	CCI	Opioids
Kim and Suh, 2023^65^	South Korea	Retrospective cohort	Jan 2010–Dec 2015	Primary and secondary	Adult outpatients using opioids for chronic non-cancer pain	CCI	Opioids
Weiner and colleagues, 2022^72^	USA	Retrospective cohort	Jan 2015–Dec 2018	Primary and secondary (population based)	Opioid-naive community dwelling adults prescribed index opioid in 2015	LTC count	Opioids
Thakarar and colleagues, 2021^70^	USA	Cross-sectional	N/R	Secondary	People with HIV on chronic opioid therapy	CCI	Opioids
Häuser and colleagues, 2020^63^	Germany	Retrospective cohort	Jan 2012–Dec 2017	Primary and secondary	Adults outpatients with chronic non-cancer pain	CCI [Quan update]	Opioids
Lobo and colleagues, 2020^67^	USA	Retrospective cohort	Jan 2007–Dec 2015	Primary and secondary	Adults patients enrolled in Medicaid prescribed an opioid	ECI	Opioids
Glanz and colleagues, 2019^62^	USA	Case control	Jan 2006–June 2018	Primary and secondary	Adult outpatients	CCI [Quan-Deyo]	Opioids
Shafi and colleagues, 2018^69^	USA	Retrospective cohort	Jan 2013–Sep 2015	Secondary	Inpatient adults who underwent surgical or endoscopic procedures	CCI	Opioids
Zhang and colleagues, 2018^73^	USA	Retrospective cohort	Jan 2011–March 2015	Secondary	Hospital inpatient adults with sepsis	CCI	Opioids
Maiti and colleagues, 2018^68^	USA	Retrospective cohort	Sep 2014–Oct 2015	Secondary	Older (>65 yr) adult inpatients in internal medical wards	CCI	Opioids
Lee and colleagues, 2016^66^	Taiwan	Retrospective cohort	2000–2011	Secondary	Female adult hospital inpatients with breast cancer	CCI	Opioids
Thorell and colleagues, 2014^71^	Sweden	Retrospective cohort	2006–2007	Primary and secondary (population based)	Total population of older adults (≥75 yr) in Östergötland County	ACG case-mix system	Opioids
Kessler and colleagues, 2013^64^	USA	Retrospective cohort	Jan 2009–Dec 2010	Secondary	Postoperative hospital inpatient adults	CCI [Dartmouth-Manitoba]	Opioids
Gianni and colleagues, 2011^61^	Italy	Non-randomised interventional	N/R	Secondary	Ambulatory older (>70 yr) outpatients with chronic non-cancer pain	Cumulative Illness Rating Scale	Opioids
Zemedikun and colleagues, 2022^74^	UK	Retrospective cohort	Jan 2000–Dec 2019	Primary	Primary care adults with OA (matched to those without OA)	LTC count	Opioids and NSAIDs^∗^
Bonnesen and colleagues, 2023^85^	Denmark	Retrospective cohort	2012–Dec 2020	Primary and secondary (population based)	Primary and secondary care patients with first time HbA1C ≥48 mmol mol^−1^	rDANCAMI score	NSAIDs
Yamanashi and colleagues, 2023^83^	USA	Retrospective cohort	Jan 2016–March 2020	Secondary	Voluntary adult inpatients or ED attenders	CCI	NSAIDs
Hall and colleagues, 2022^80^	USA	Retrospective cohort	Sep 2012–May 2018	Secondary	Older (≥60 yr) surgical trauma inpatients	LTC count	NSAIDs
Glassou and colleagues, 2019^79^	Denmark	Prospective cohort	Jan 2005–Dec 2016	Secondary	Older (≥65 yr) adult inpatients with a surgically treated hip fracture	CCI	NSAIDs
Cho and colleagues, 2018^77^	South Korea	Case control	Jan 2010–Dec 2013	Primary and secondary	South Asian outpatient adults with rheumatoid arthritis	CCI	NSAIDs
Liu and colleagues, 2018^81^	Canada	Case control	July 1991–March 2015	Secondary	Adult inpatients hospitalised with TMA	ADG score	NSAIDs
Gallagher and colleagues, 2012^78^	USA	Retrospective cohort	N/R	Secondary	Adult hospital inpatients or outpatients with depression	CCI [age adjusted]	NSAIDs
Humes and colleagues, 2011^75^	UK	Case control	Jan 1990–Dec 2005	Primary care	Patients with diverticular perforation	CCI	NSAIDs
Abraham and colleagues, 2008^76^	USA	Retrospective cohort	Jan 2000–Dec 2002	Primary & secondary	Older adult (>65 yr) veteran outpatients	CCI [Deyo] and LTC count	NSAIDs
Stockl and colleagues, 2005^82^	USA	Retrospective cohort	July 1998–Aug 2002	Primary and secondary	Adult outpatients	CCI	NSAIDs
Park and colleagues, 2022^84^	USA	Retrospective cohort	Jan 2009–May 2022	Secondary	Older (>65 yr) postoperative adult hospital inpatients	Combined comorbidity index	Gabapentinoids

### Multimorbidity measurement

A definition of multimorbidity was provided in one study,^74^ but otherwise, multimorbidity was not defined explicitly in the remaining studies. Multimorbidity was measured using the following indices; the Charlson Comorbidity Index (CCI) or modification (*n*=17),^60^^,^^62^, ^63^, ^64^, ^65^, ^66^^,^^68^, ^69^, ^70^^,^^73^^,^^75^, ^76^, ^77^, ^78^, ^79^^,^^82^^,^^83^ LTC count (*n*=4),^72^^,^^74^^,^^80^^,^^86^ Elixhauser comorbidity index (ECI, *n*=1),^67^ combined comorbidity index (*n*=1),^84^ cumulative illness rating scale (*n*=1),^61^ Danish Comorbidity Index for Acute Myocardial Infarction restricted to non-cardiovascular diseases (rDANCAMI) score (*n*=1),^85^ John Hopkins aggregated diagnosis group score (ADG) (*n*=1)^81^ or adjusted clinical group (ACG) case-mix system.^71^ Most studies did not report the number of conditions incorporated in their reported measure of multimorbidity.^60^, ^61^, ^62^^,^^65^^,^^67^, ^68^, ^69^, ^70^, ^71^, ^72^, ^73^^,^^75^, ^76^, ^77^, ^78^, ^79^, ^80^, ^81^, ^82^, ^83^, ^84^^,^^86^

### Assessment of heterogeneity

Quantitative assessment of heterogeneity was not possible because of the absence of quantitative synthesis. A formal qualitative assessment of heterogeneity was not judged to be of value based on the marked variation in study methodologies, analgesic exposure(s), adverse outcome(s), measurement and reporting of multimorbidity.

### Risk of bias

Risk of bias of observational studies included in the review is summarised in Table 2. Overall, 10 studies were rated as ‘*good*’, six ‘*fair*’, and 10 ‘*poor*’. Few studies used a new user design with quantification of time-varying cumulative analgesic exposure. Other common sources of bias included measurement and misclassification through an absence of analgesic dosing informationor adequate description, length or completeness of follow-up. Quality assessment of the single interventional study is summarised in Supplementary material S3. It was judged to be at serious risk of bias because of the absence of both a comparator group and multivariable adjustment.

**Table 2:** Risk of bias of observational studies.

Author year	Selection	Comparability	Outcome/exposure	Overall
Cohort studies				
Kim 2023	2∗	1∗	2∗	Fair quality
Bonnesen 2023	2∗	2∗	2∗	Fair quality
Lee 2016	2∗	2∗	2∗	Fair quality
Lobo 2020	2∗	2∗	2∗	Fair quality
Park 2022	2∗	2∗	2∗	Fair quality
Stockl 2005	2∗	2∗	2∗	Fair quality
Abraham 2008	3∗	2∗	2∗	Good quality
Glassou 2019	3∗	2∗	2∗	Good quality
Häuser 2020	4∗	2∗	2∗	Good quality
Shafi 2018	3∗	1∗	2∗	Good quality
Weiner 2022	3∗	2∗	2∗	Good quality
Zemedikun 2022	3∗	1∗	2∗	Good quality
Zhang 2018	3∗	1∗	2∗	Good quality
Cano-Escalera 2023	1∗	2∗	2∗	Poor quality
Gallagher 2012	2∗	1∗	1∗	Poor quality
Hall 2022	0∗	2∗	0∗	Poor quality
Kessler 2013	1∗	1∗	2∗	Poor quality
Maiti 2018	3∗	0∗	2∗	Poor quality
Thorell 2014	3∗	2∗	1∗	Poor quality
Yamanashi 2023	1∗	2∗	1∗	Poor quality
Case control studies				
Cho 2018	4∗	2∗	1∗	Poor quality
Podesser 2024	2∗	1∗	1∗	Poor quality
Glanz 2019	4∗	2∗	2∗	Good quality
Humes 2011	4∗	2∗	2∗	Good quality
Liu 2018	4∗	2∗	2∗	Good quality
Cross-sectional studies				
Thakarar 2021	0∗	2∗	1∗	Poor quality

### Opioids

Among 16 studies that investigated opioids, 12 reported evidence of harms, while four found no evidence of harm. Characteristics and results of studies reporting adverse opioid-related outcomes are summarised in Table 3.

**Table 3:** Characteristics and results of studies of opioids, *n*=16. ACG, adjusted clinical group; ADE, adverse drug event; ADR, adverse drug reaction; aHR, adjusted hazard ratio; AIDS, acquired immunodeficiency syndrome; anova, analysis of variance; ARR, adjusted rate ratio; BZD, benzodiazepine; CCI, Charlson Comorbidity Index; CI, confidence interval; CIRS, cumulative illness rating scale; CHF, congestive heart failure; DM, diabetes mellitus; DVT, deep venous thrombosis; ECI, Elixhauser comorbidity index; ED, emergency department; EHR, electronic health record; HIV, human immunodeficiency virus; HR, hazard ratio; JH, John Hopkins; ICD, International Classification of Diseases; IMRD-UK, IQVIA Medical Research Data UK; IQR, interquartile range; LCA, latent class analysis; LHID-CIP, Longitudinal Health Insurance Database for Catastrophic Illness Patients; LOS, length of stay; LTC, long-term condition; MEQ, morphine equivalent; MM, multimorbidity; MME, morphine milligram equivalent; MNA, Mini Nutritional Assessment; NHIS-NSC, National Health Insurance Service National Sample Cohort; N/R, not reported; OA, osteoarthritis; OD, overdose; ORADE, opioid-related adverse drug event; OR, odds ratio; PPI, proton pump inhibitor; PRN, as required; PSM, propensity score matching; RUB, resource utilisation bands; sd, standard deviation; WBC, white blood cell. ∗Age in yr presented as mean (SD) unless stated otherwise. ^†^Patients in the same ACG group have the ‘same type and degree of multimorbidity’.

Studies reporting drug-related harm (secondary care)								
Reference	Sample size	Patient characteristics			Analgesic exposure	Adverse outcome definition and timing	Analysis	Main findings
		Measurement of multimorbidity	Female, *n* (%)	Age∗				
Shafi and colleagues, 2018^69^	135 379	CCI measured upon admission via ICD-9. Number of conditions N/R. Mean (sd) CCI sub-populations: ORADE 4.0 (2.9) *vs* non-ORADE 2.2 (2.7).	91 371 (67.5)	58.9 (17.4)	Administrative database. Prevalent user design. No information on dosing.	ORADE defined as one or more well-known adverse effects of opioids during hospitalisation.	Unadjusted differences between ORADE and non-ORADE.	*MM measure higher in ORADE group:* ORADE positive (*n*=4386, 10.6%) mean (sd) CCI 4.0 (2.9) *vs* ORADE negative (*n*=120 993) CCI 2.2 (2.7). *MM measure higher with increasing ORADE severity:* CCI mean (sd): Severe ORADE: 4.3 (2.8); moderate: 3.8 (2.9); mild: 3.1 (2.8).
Kessler and colleagues, 2013^64^	37 031	CCI [Dartmouth-Manitoba] measured during hospitalisation via ICD-9. 19 conditions in MM measure. Mean (sd) CCI study population: 1.15 (1.7) [pre-PSM].	25 403 (68.6)	60.4 (18.3)	Charge codes from administrative data of financial transactions. Prevalent user design. No information on dosing.	ORADE defined as per large list in Table 2 during hospitalisation.	Multiple logistic regression with PSM 1:1 age, race-ethnicity, sex, pre-surgery opioid use, and comorbidities. Adjusted for age ≥65 yr, sex, race-ethnicity, obesity, degenerative joint disease, and opioid use before surgery.	*MM measure higher in ORADE group*: ORADE (*n*=4955, 13.6%) mean (sd) CCI 1.87 (2.1) *vs* no ORADE (*n*=31 574) CCI 1.04 (1.6) [pre-PSM]. *Increasing MM measure associated with ORADEs among opioid exposed adults:* Risk of ORADEs by CCI (per one point increase) OR 1.18 (95% CI 1.16–1.19, *P*<0.0001).
Zhang and colleagues, 2018^73^	5994	CCI. Timing and number of conditions N/R. Mean (sd) CCI sub-population, opioid exposed group: 4.67 (5.71).	3,006 (50.1)	60.9 (N/R)	Medication administration chart during hospitalisation. New user design. No information on dosing.	All-cause mortality at 28 days.	Cox proportional hazard analysis (1) unadjusted; (2) age, gender, BMI, WBC count.	*Opioids use vs non-use associated with mortality in MM adults:* Crude mortality: opioid 10.35% *vs* non-opioid 2.4%. Unadjusted HR 5.951 (4.218–8.396) <0.0001; demographic model (2) aHR 7.321 (5.178–10.349) <0.0001.
Maiti and colleagues, 2018^68^	9245	CCI. Timing and number of conditions N/R. Mean (sd) CCI study population: 7.1 (2.5).	5099 (55.2)	80.3 (8.8)	EHR prescribing data with morphine milligrams equivalent per patient. Non-, prior and new opioid users recorded.	Hospital length of stay and 30-day hospital re-admission.	Descriptive/anova without adjustment.	*Current and former opioid users have longer LOS and 30-day readmissions than non-users in MM adults:* new opiates (*n*=1915): MEQ per patient, mean (sd) 296.9 (1779.1). LOS mean 7.7 (0.2) *P*<0.001. 30-Day readmissions 426 (22.3%). Prior opiates (*n*=400): MEQ per patient, mean (sd) 421.9 (1948.3). LOS mean 6.8 (sd 0.3). 30-day readmissions 100 (25.0%). No opiates (*n*=6930). LOS mean 5.2 (sd 0.1). 30-day readmissions 1361 (19.6%). Participants never exposed to opiates had a shorter LOS than those receiving short- and long-acting opiates (5.2, 7.3, 8.6 days; *P*<0.001) and less likely to be readmitted within 30-days (19.6%, 27.7%, 28.9%; *P*<0.001).
Gianni and colleagues, 2011^61^	93	CIRS measured at baseline. Number of conditions N/R. Mean CIRS of study population 3.4 (i.e. severe comorbidities in three organ categories) with mean index of severity 1.8 (i.e. low grade).	66 (71)	79.7 (7.1)	Primary data collection at study visits. Buprenorphine transdermal delivery system (TDS) started using a dose calculated based on equivalent analgesic dose ratios. New user design.	ADE categorised as slight *vs* moderate *vs* severe (unclear how defined), cessation of treatment and study withdrawal because of side-effects. Nausea, constipation, sleepiness, rash measured up to 90 days.	Descriptive/ANOVA without adjustment.	*Opioids associated with ADEs in MM adults*: n=44 (47.3%) reported ADEs; *n*=39 (89%) slight or moderate clinical intensity and *n*=5 (11%) severe. *n*=35 (37%) of patients stopped treatment prematurely; *n*=12 (12.9%) withdrew because of side-effects. The systemic effects most frequently noted: Nausea *n*=7 (15.7%), constipation *n*=7 (15.7%), sleepiness *n*=6 (14.2%), rash *n*=5 (11.2%).
Thakarar and colleagues 2021^70^	153	CCI measured at baseline. Number of conditions N/R. Mean/median CCI N/R.	54 (35)	54 median (IQR 49–59)	Prescription fill data. MME measured at baseline. Prevalent user design.	ED attendance at 1 yr.	Multivariate logistic regression adjusted for age, gender, years on opioids, high-dose opioids, co-prescribing of BZD, lack of opioid treatment agreement, hepatitis C, depression, prior ED visits, HIV-1 RNA <200 copies ml^−1^.	*Increasing MM measure associated with ED attendance among opioid exposed adults:* CCI (per one point increase) OR 1.47 (95% 1.17–1.85) *P* 0.001.
***Studies reporting drug-related harm (primary and secondary care)***								
Weiner and colleagues, 2022^72^	236 921	LTC count based on Elixhauser comorbid conditions measured 2 yr before index prescription using ICD-9 and ICD-10. Number of conditions N/R. Compared 1–2 *vs* 0 and ≥3 *vs* 0 LTCs.	133 860 (56.5)	N/R (age category only)	Prescription fill data from linked administrative claims and public health datasets. Monthly time-varying MME sum and number of opioid fills calculated cumulatively over 6 months. New user design.	Opioid overdose occurring anytime during follow-up (max 4 yr).	Cox proportional hazard analysis adjusted for index prescription, long-acting formulation, number of benzodiazepine prescriptions, opioid and benzodiazepine overlap, sum of MME, prescription from ≥3 prescribers or pharmacies.	*Opioid overdose increases when stratified by MM measure:* LTC count, *n* (%): 0: 130 093 (54.9%). OD per 100 000 person yr 73.2 (95% CI 64.1–83.6). LTC 1–2: 84 018 (35.5%). OD per 100 000 person yr 138.2 (95% CI 122.9–155.4). LTC 3+: 22 810 (9.6%). OD per 100 000 person yr 321.9 (95% CI 276.8–374.3) *MM measure associated with opioid overdose in opioid exposed adults:* HR by LTC count: 0: Reference; 1–2: 1.32 (95% CI 1.08–1.62) *P* 0.01; ≥3: 1.90 (95% CI 1.42–2.53) *P*<0.001.
Thorell and colleagues, 2014^71^	38 407	JH ACG case-mix grouped by RUBs.^†^ measured from 2006. Number of conditions N/R. Compared subgroups with (RUBs 3–5) and without (RUBs 0–2) MM.	23 428 (61%)	N/R (age category only)	Administrative healthcare data. Prevalent user design. No information on dosing.	Hip fracture occurring any time up to 1 yr.	Multiple logistic regression: Model A (unadjusted), B (age), C (age and gender) to D (age, gender and MM level).	*Opioids associated with hip fracture in a subgroup of adults with MM compared with those without MM:* opioids RUBs 0–2: OR 1.45 (0.99–2.11) *P*>0.05 *vs* RUBs 3–5: OR 1.71 (1.44–2.03) *P*<0.05 [model C].
Kim and Suh, 2023^65^	22 524	CCI. Measured 1 yr before index. Number of conditions N/R. Mean (sd) CCI study population 2.0 (sd 1.8). Cohort stratified by CCI 0: 20.8%; 1: 26.0%; 2: 20.9%; ≥3: 32.3%.	14 641 (65)	64 (13)	Prescription fill data based on NHIS-NSC database. Daily dose of opioids prescribed on index date used to calculate MME. Prevalent user design.	Opioid abuse defined as shopping or prescription overlap anytime during follow-up (max 6 yr).	Cox proportional hazard analysis adjusted for age, sex, type of health insurance, history of alcohol abuse, daily dose of opioids prescribed on the index date, a history of opioid abuse categorised as 0, 1–2, and ≥3 of previous abuse events.	*MM measure associated with opioid abuse among opioid exposed adults*: Prescription opioid abuse by baseline characteristics CCI (per one point increase) 1.054 (95% CI 1.044–1.065) *P*<0.0001.
Glanz and colleagues, 2019^62^	14 898	CCI [Quan-Deyo modified]. Timing of MM assessment N/R. Number of conditions N/R. Mean (sd) CCI study sub-populations cases 2.2 (2.3) *vs* controls 1.4 (1.8).	8,983 (60.3)	N/R (age category only)	Pharmacy claims data using national drug codes. MME calculated for every month of follow-up. Prevalent user design.	Opioid overdose anytime during follow-up (max 6 yr).	Multivariate logistic regression. Each case patient with overdose was matched with 20 controls adjusted for opioid dose in the 3 months before index, age, sex, receipt of extended-release, long-acting, or both opioid, mental health disorder, drug or alcohol use disorder, benzodiazepine dispensings, tobacco use or use disorder and race/ethnicity.	*MM measure associated with opioid overdose among opioid exposed adults*: Modified CCI (per one point increase): Unadjusted OR 1.21 (95% CI 1.14–1.28); adjusted OR 1.25 (95% CI 1.16–1.35). Adjusted OR for opioid overdose for those that had sustained opioid discontinuation (i.e. 3-month cessation) modified CCI 1.26 (95% CI 1.17–1.36).
Häuser and colleagues, 2020^63^	6464	CCI [Quan update] measured at baseline. 14 conditions to establish. Mean/median CCI N/R.	3620 (56)	66.3 (16.6)	Prescription fill data from administrative database. Opioid dosage from MEQ values (time-varying), with annual recalculations during follow-up. New user design.	All-cause mortality anytime during follow-up (max 60 months).	PSM and cox proportional hazard models adjusted for age, gender, quarter of index treatment, estimated propensity score, study opioid cohort, and treatment duration.	*MM measure associated with mortality among adults prescribed opioids*: Subgroup with <100 MEQ day^−^^1^ (*N*=2943): CCI (per one point increase): HR 1.19 (1.16–1.22) *P*<0.0001. Subgroup with >100 MEQ day^−^^1^ (*N*=189): CCI 1.19 (1.16–1.23) *P*<0.0001.
***Studies reporting drug-related harm (primary care)***								
Zemedikun and colleagues, 2022^74^	661 499	Measured by LTC count any time before diagnosis of OA based on 30 diseases. LTC median (IQR) study sub-populations OA group LTC 2 (1–4) *vs* non-OA group LTC 1 (0–3).	396 802 (60.0)	62.8 (12.4)	Prescription data from IMRD-UK EHR database. Prevalent user design. No information on dosing.	All-cause mortality anytime during follow-up post lag period of 1 yr from index date (maximum 9 yr).	Cox proportional hazard analysis with LCA matched 1:1 (based on age, sex, and general practice) adjusted for comorbidity phenotypes, age, sex, BMI categories, deprivation quintiles, smoking status, and ethnicity.	*Opioids associated mortality across all groups, but with greater strength among MM patients*: Cases (i.e. MM): no opioid (reference); weak opioids aHR 1.18 (95% CI 1.15–1.21); strong opioids aHR 1.80 (95% CI 1.69–1.92). Controls (i.e. non-MM): no opioid (reference); weak opioids 1.34 (95% CI 1.29–1.39); strong opioids 1.68 (95% CI 1.50–1.89).
***Studies not reporting drug-related harm (any setting)***								
Lobo and colleagues, 2019^67^	432 110	ECI measured 6 months before index opioid. Number of conditions N/R for ECI which was 'modified by removing the conditions included as covariates'. Mean/median ECI N/R.	291 674 (67.5)	30.7 (11.1)	Administrative database. New user design. Dose captured.	Opioid use disorder (ICD-9/10); opioid overdose; opioid misuse (based on number of unique prescribers and pharmacies) anytime during study period (maximum 9 yr).	Multivariate logistic regression with adjustment for age, race, urban/rural living area, enrolment characteristics (eligibility category, managed care/fee-for-service), baseline comorbid conditions (alcohol abuse/dependence, non-opioid drug abuse/dependence, adjustment mood or anxiety disorders, back or neck pain, arthritis/joint pain, headache/migraine, HIV/AIDS), baseline use of benzodiazepines, neuromuscular blocking agents, and ED visits.	*Impact of MM measure on drug-related harms among opioid exposed adults:* Opioid use disorder: ARR by index prescriber: ECI 0.87 [95% CI 0.85–0.88] *P*<0.0001. ARR by dominant prescriber: ECI 0.87 [95% CI 0.86–0.89] *P*<0.0001. Overdose: ARR by index prescriber: ECI 0.97 [95% CI 0.92–1.02] *P* 0.26. ARR by dominant prescriber: ECI 0.98 [95% CI 0.93–1.03] *P* 0.360. Opioid misuse: ARR by index prescriber ECI 0.97 [95% CI 0.96–0.99] *P* 0.004. ARR by dominant prescriber ECI 0.97 [95% CI 0.95–0.98] *P*<0.0001.
Lee and colleagues, 2016^66^	73 917	CCI measured at baseline. 12 Conditions to calculate CCI. Study population CCI 0: 91.9%, 1–2: 6.79%, ≥3: 1.28%. Study sub-populations: morphine group CCI 0: 90.2%, 1–2: 8.21%, ≥3: 1.56%; no morphine group CCI 0: 92.5%, 1–2: 6.32%, ≥3: 1.18%.	73,917 (100)	52.3 (11.9)	Insurance claims data from LHID-CIP national administrative database to dichotomise users *vs* non-users of morphine. No information on dosing. New user design.	Atrial fibrillation anytime during follow-up (maximum 10 yr).	Cox proportional hazard analysis adjusted for age, hypertension, hyperlipidaemia, hyperthyroidism, bisphosphonate use and tamoxifen use.	*Association between morphine use and atrial fibrillation stratified by MM measure:* in morphine users *vs* non-users, HR stratified by CCI 0: 4.60 (95% CI 3.62–5.84) *P*<0.001, 1–2: 4.39 (95% CI 2.79–6.90) *P*<0.001, ≥3: 2.50 (95% CI 1.09–5.73) *P*<0.05.
Podesser and colleagues, 2024^86^	74	LTC count. Unclear when measured. Number of conditions N/R. LTC study population 7.24 (3.6). LTC count cases 7.4 (3.9). LTC controls 7.0 (3.2)	28 (37.8)	82.6 (5.8)	Medical administration record to determine regular *vs* PRN use of hydromorphone or fentanyl. Prevalent user design. No information on dosing.	Falls during inpatient hospitalisation excluding those within 48 h of admission.	Binomial logistic regression analyses through matched case control study matched for sex, age, length of stay, and severity of illness.	*Opioids were not associated with falls in adults with MM:* 0–24 h before fall: opioid intake (0–1) OR 2.24 (95% CI 0.33–15.18) *P* 0.410. 24–48 h before fall: opioid intake (0–1) OR 7.44 (0.70–78.65) *P* 0.095.
Cano-Escalera and colleagues, 2023^60^	714	CCI. Timing and number of conditions N/R. Study population CCI mean (sd) 6.39 (2)	346 (48.5)	84.37 (6.76)	EHR. Prevalent user design. No information on dosing.	All-cause mortality up to 2 yr.	Cox proportional hazard analysis adjusted for age, sex, weight, own-home, Barthel, Pfeiffer, MNA weight loss 3 months, MNA mobility, MNA acute disease 3 months, vision loss, constipation, falls, CHF, DVT, cerebrovascular disease, DM, thyroid disease, drug oligopharmacy, PPIs, zolpidem, antidiabetics, and diuretics.	*Opioids associated with a reduction in mortality in sub-population of MM adults:* pre-frail sub-population only: opiates HR 0.000069 [95% CI 0.00000035–0.013, *P*<0.001]

#### Evidence of drug-related harm (n=12)

##### Secondary care settings (n=6)

Six studies reported opioid-related harms in secondary care settings including opioid-related adverse drugs events (ORADEs), hospital length of stay, 30-day re-hospitalisation, emergency department (ED) attendance and mortality.^61^^,^^64^^,^^68^, ^69^, ^70^^,^^73^ Two studies reported a greater prevalence of multimorbidity (i.e. a higher mean CCI) among adults who experienced postoperative ORADEs.^64^^,^^69^ CCI was reported to have a dose–response relationship with the severity of ORADEs^69^ and a modest predictive effect of ORADEs in postoperative adults.^64^ Two studies in medical inpatient settings reported adverse outcomes associated with opioid use in multimorbid populations of adults.^68^^,^^73^ Opioid exposure was associated with a seven-fold increase in mortality compared with non-use among patients with sepsis after adjustment for demographic, but not clinical factors.^73^ Among older adults (>65 yr), current and former opioid users had a longer hospital length of stay and an increase in 30-day readmissions compared with non-users on descriptive analyses.^68^ Two studies were conducted in outpatients.^61^^,^^70^ In a small trial of buprenorphine for a multimorbid population of older adults with chronic non-cancer pain, nearly half of patients reported adverse events including nausea, constipation, sleepiness, and rash. The findings were descriptive and based on a select cohort of volunteers.^61^ Finally, CCI was associated with ED attendance among patients with human immunodeficiency virus (HIV) treated with chronic opioid therapy.^70^

##### Primary and secondary care settings (n=5)

Five studies conducted in primary and secondary care reported opioid-related harms (i.e. opioid overdose, hip fracture, opioid abuse, and mortality).^62^^,^^63^^,^^65^^,^^71^^,^^72^ Two studies used population-based data to compare subgroups of people with and without multimorbidity, reporting increased risk of adverse outcomes in individuals with multiple LTCs.^71^^,^^72^ Multimorbidity (i.e. increasing LTC count) was associated with an increased incidence of opioid overdose after first opioid prescription for pain.^72^ Opioids were associated with an increased risk of hip fracture in a subgroup of adults with multimorbidity (i.e. resource utilisation bands [RUB] level 3–5), but not those without (i.e. RUB level 0–2) in a regression analysis adjusted for age and gender. RUB groups are a proxy indicator of multimorbidity based on the John Hopkins ACG case-mix system.^71^ Multimorbidity (i.e. increasing CCI) was associated with a marginal increase in opioid abuse^65^ and modest increase in overdose^62^ and mortality^63^ among adults exposed to long-term opioids for chronic non-cancer pain.^62^^,^^63^^,^^65^

##### Primary care settings (n=1)

One study, conducted using primary care data, assessed the impact of opioids and NSAIDs on mortality in a population of adults with osteoarthritis and multimorbidity compared with matched controls (without multimorbidity). The authors reported an increase in mortality with weak and strong opioids across all groups, but more so for strong opioids in the sub-population of adults with multimorbidity.^74^

#### No evidence of drug-related harm (n=4)

Four studies did not report evidence of adverse opioid-related outcomes (i.e. opioid use disorder, opioid misuse, opioid overdose, atrial fibrillation, falls or mortality) associated with multimorbidity, or, opioid exposure among people with multimorbidity.^60^^,^^66^^,^^67^^,^^86^ One study found a higher disease burden (measured using the ECI) was associated with a relative risk reduction in opioid use disorder and misuse, but not overdose.^67^ One study reported an association between morphine use and atrial fibrillation compared with non-use among female breast cancer patients, with stratification by CCI showing that the observed association was attenuated with increasing multimorbidity.^66^ Two small, single-centre studies assessed the impact of opioid use compared with non-use among populations of older adults with multimorbidity.^60^^,^^86^ One reported no association between short-term opioid intake (0–24 or 24–48 h) and falls.^86^ The other reported a dramatic relative reduction in mortality associated with opioid use (defined only as use *vs* non-use) in a pre-frail sub-population of adults, however, crude mortality was not reported.^60^

### NSAIDs

Among 11 studies that investigated NSAIDs, six reported evidence of harms, while five found no evidence of harm. Characteristics and results of studies reporting adverse NSAID-related outcomes are summarised in Table 4.

**Table 4:** Characteristics and results of studies of NSAIDs, *n*=11. AKI, acute kidney injury; aOR, adjusted odds ratio; CAD, coronary artery disease; CAM-ICU, Confusion Assessment Method for Intensive Care Unit; CCI, Charlson Comorbidity index; CHF, congestive heart failure; CI, confidence interval; CKD, chronic kidney disease; Cox-2i, Cox-2 selective inhibitor; CV, cardiovascular; CVA, cerebrovascular accident (stroke); CVD, cardiovascular disease; DM, diabetes mellitus; DOSS, Delirium Observation Screening Scale; DRS-R-98, Delirium Rating Scale-Revised-98; EHR, electronic health record; GI, gastrointestinal; GPRD, General Practice Research Database; HIV, human immunodeficiency virus; HR, hazard ratio; HRA, Health Insurance Review and Assessment; HTN, hypertension; IMRD-UK, IQVIA Medical Research Data UK; IQR, interquartile range; JH ADG, John Hopkins Aggregated Diagnosis Group Score; KDIGO, kidney disease improving global outcomes; LCA, latent class analysis; LTC, long-term condition; MI, myocardial infarction; MM, multimorbidity; N/R, not reported; (ns) NSAIDs, (nonselective) nonsteroidal anti-inflammatory drugs; OA, osteoarthritis; OR, odds ratio; PSM, propensity score matching; RA, rheumatoid arthritis; RBC, red blood cell; rDANCAMI, Danish Comorbidity Index for Acute Myocardial Infarction restricted to non-cardiovascular diseases; RR, relative risk; sCr, serum creatinine; sd, standard deviation; SLE, systemic lupus erythematous; SNRI, serotonin–norepinephrine reuptake inhibitor; SSRI, selective serotonin reuptake inhibitor; TMA, thrombotic microangiopathy; UGI, upper gastrointestinal. ∗Age in yr presented as mean (sd) unless stated otherwise. ^†^Exposure of interest=i.v. ketorolac hence very unlikely to be any prevalent users. ^‡^i2b2 cohort only.

*Studies reporting drug-related harm (secondary care)*								
Reference	Sample size	Patient characteristics			Analgesic exposure	Adverse outcome	Analysis	Main findings
		Measurement of multimorbidity	Female *n* (%)	Age∗				
Glassou and colleagues, 2019^79^	74 791	CCI measured over 10 yr before hip fracture. Number of conditions N/R. Outcomes stratified according to MM measure (low/medium/high burden groups based on CCI 0–1 *vs* 2 *vs* ≥3).	53 337 (71.3)	N/R (age category by subgroup only).	Community dispensation data. Current users (≥1 redeemed prescription within 90 days of hip fracture surgery), former users (≥1 prescription 91–365 days and no prescriptions within 90 days of the hip fracture surgery) *vs* non-users. No dosing information. Prevalent user design.	Postoperative RBC transfusion within 7 days of hip fracture surgery.	Cumulative incidence with competing risk of death and log-binomial model adjusted for age, sex, type of fracture, surgery delay, year of surgery, other drug classes, and department.	*NSAIDs associated with postoperative RBC transfusion in adults with MM:* blood transfusion within 7 days of surgery stratified by MM measure, cumulative incidence, and RR. Current NSAID use *vs* non-use: CCI 0–1: 43.2% (95% CI 41.6–44.8%). RR 1.07 (1.02–1.12); CCI 2: 48.0% (95% CI 46.3–49.7%). RR 1.06 (1.01–1.11); CCI ≥3: 54.0% (95% CI 51.5–56.5%). RR 1.09 (1.04–1.15).
Gallagher and colleagues, 2012^78^^‡^	1528	CCI [age adjusted]. Timing and number of conditions N/R. Mean CCI study sub-populations (NSAID exposed 6 *vs* non-exposed 2). Median CCI (IQR) study sub-populations (NSAID 6 [6] vs non-exposed 2 [5])	1084 (70.9)	51.8 (15.9)	≥1 Documented prescription during classified antidepressant treatment period from EHR. Split into chronic (>2 NSAID prescriptions or refills at a daily dose within the study period) or intermittent users. No dosing information. Prevalent user design.	Treatment-resistant depression defined as remaining depressed despite two or more antidepressant treatments. Timing and follow-up N/R.	Multivariate logistic regression adjusted for age, sex, race, and insurance payer status (model 1).	*Non-selective NSAIDs associated with treatment-resistant depression in adults with MM:* ns NSAIDs only: unadjusted OR: 1.74 (95% CI 1.40–2.18); model 1: OR 1.56 (95% CI 1.23–1.97). Cox2i: Unadjusted OR 0.83 (95% CI 0.61–1.12); model 1: OR 0.89 (95% CI 0.65–1.22). Salicylates: Unadjusted OR 1.01 (95% CI 0.82–1.25); model 1: OR 1.15 (95% CI 0.91–1.47).
Hall and colleagues, 2022^80^	316	LTC count. Timing and number of conditions included N/R. Median (IQR) number of LTCs among patients with AKI 1 (0.25–2) *vs* non-AKI 0 (0–0).	191 (60)	73 (10)	Exposure definition and measurement N/R. Ketorolac administration, was at the ‘discretion of the provider’ (limited to 5 days use). Dose recorded. Prevalent user design^†^.	Acute kidney injury defined via KDIGO (i.e. increase in sCr >0.3 mg dl^−1^ within 48 h, or an increase to 1.5 times baseline within the prior 7 days that persists for at least 48 h).	Multivariable logistic regression and classification and regression tree analysis adjusted for base: loop diuretics, radiocontrast, vasopressors, total number of nephrotoxins, (total number of comorbidities), and average daily ketorolac dose for comparison. Final model: Loop diuretics.	*MM measure associated with AKI among NSAID exposed adults*: OR base model: Number of comorbidities 3.2 (1.3–7.7) *P* 0.01. aOR final model: Number of comorbidities OR 2.4 (1.1–5.2) *P* 0.021. AKI 8/316 (2.5%). AKI patients: Median ketorolac dosing cumulative=37.5 (15–71) mg. Mean daily dose ketorolac=17.5 (5.3) mg. Median ketorolac doses=2 (1–4.5). Median duration ketorolac=1 (1–2) days.
***Studies reporting drug-related harm (primary and secondary care)***								
Abraham and colleagues, 2008^76^	474 495	CCI [Deyo] and LTC count. Measured within 1 yr before index prescription. Number of conditions N/R. LTC count of study population: 0–1: 76.4%; ≥2: 23.6%.	9964 (2.1)	73 (5.5)	Prescription fill data via national administrative prescription database. All NSAIDs at ‘full musculoskeletal dose’. New user design.	All-cause mortality in the 365 days among the older patients who had experienced a UGI endoscopy, MI or CVA after index NSAID prescription.	Cox proportional hazard analysis with adjustment for age, gender, steroid use, statin use, SSRI use, SNRI use, antianginal use, UGI endoscopy, MI, CVA, history of MI, liver disease, diabetes (type N/R), CHF, CKD, and rheumatological disease.	*Crude mortality greater in adults with MM compared with those without MM among NSAID users*: 6290 died while exposed to an NSAID (i.e. current users). Of those who died, patients with MM (LTC ≥2) 46.3% *vs* patients without MM (LTC 0–1) 53.7% (*P*<0.001). *MM measure associated with mortality among NSAID exposed adults*: Deyo comorbidity index: 1 HR 1.7 (95% CI 1.5–1.7); 2 HR 2.2 (95% CI 2.1–2.4); 3–4 HR 2.9 (95% CI 2.9 CI 2.7–3.2); ≥4 HR 3.7 (95% CI 3.2–4.4).
Stockl and colleagues, 2005^82^	70 014	CCI. Measured during the 6 month pre-period. Number of conditions included N/R. CCI study sub-populations CCI mean (sd) cox-2i group 0.29 (0.95) *vs* ns NSAID group 0.3 (0.98).	45 789 (65.4)	63.4 (16.3)	Prescription fill data based on pharmacy codes from administrative claims database. Cox-2i or ns NSAIDs. No dosing information. New user design.	Inpatient hospitalisation for GI bleeding at 1 yr.	Cox proportional hazards analysis. PSM Cox-2 and non-selective-NSAID initiators matched by age, gender, geographical state, (comorbidity index), steroid use, warfarin use, arthritis indication, history of recent GI bleed. Adjusted for age, gender, state of health plan, pre-period corticosteroid/warfarin use, and pre-period GI bleed/RA/OA indication.	*MM measure associated with GI bleeding among NSAID exposed adults:* total population: HR CCI 1.11 (95% CI 1.04–1.18). Low-risk sub-population (*n*=29 547) HR 1.26 (1.08–1.49); high-risk sub-population (i.e. age >65 yr, recent warfarin or steroid, recent hospitalisation for GI bleeding) (*n*=40 467) HR 1.10 (95% CI 1.03–1.17). *MM measure associated with GI bleeding after adjustment for pre-period use of a gastroprotective agent:* total population: HR CCI 1.10 (95% CI 1.04–1.17). Low-risk population: HR CCI 1.30 (95% CI 1.11–1.51). High-risk population: HR CCI 1.09 (95% CI 1.02–1.17).
Cho and colleagues, 2018^77^	34 120	CCI measured within 1 yr before RA index date. Number of conditions N/R. Mean (sd) CCI study population 3.9 (2.3). Median (IQR) CCI study population 2 (IQR 2–5).	26 338 (77.2)	61.4 (10.9)	NSAID (ns *vs* Cox-2i) exposure defined as use for ≥30 days in the year before CVD developed from HRA national administrative health insurance database. No dosing information. Prevalent user design.	Cardiovascular disease (i.e. composite of coronary artery disease, haemorrhagic stroke, ischaemic stroke, and peripheral artery disease).	Multivariate logistic regression. Each case was matched to up to 4 controls for age, sex, RA index date, comorbidities (hypertension, DM, hyperlipidaemia, CKD), and drugs (antiplatelet agents and cholesterol-lowering agents).	*NSAIDs associated with CV disease in adults with MM:* ns NSAIDs OR 1.32 (95% CI 1.22–1.41, *P*<0.01). Cox-2 i OR 1.31 (95% CI 1.18–1.45, *P*<0.01). Simultaneous ≥2 NSAIDs OR 1.84 (95% CI 1.66–2.05, *P*<0.01). *Ns NSAIDs and Cox2i associated with ischaemic stroke in adults with MM*: ns NSAIDs (OR 1.36, 95% CI 1.26–1.48), Cox-2i (OR 1.33, 95% CI 1.19–1.49), and simultaneous ≥2 NSAIDs (OR 1.96, 95% CI 1.74–2.20). *Simultaneous use of ≥2 NSAIDs associated with haemorrhagic stroke in adults with MM:* simultaneous ≥2 NSAIDs OR 1.72 (95% CI 1.27–2.33). *NSAIDs were not associated with CAD in adults with MM*: ns NSAIDs (OR 1.17, 95% CI 0.97–1.43), Cox-2i (OR 1.23, 95% CI 0.95–1.60).
***Studies not reporting drug-related harm (any setting)***								
Bonnesen and colleagues, 2023^85^	103 308	rDANCAMI score. Measured based on a 5-yr look back window from cohort entry. 20 Conditions included in MM measure. rDANCAMI of study population: 0: 67%, 1–3: 22%, 4–5: 4.2%, ≥6: 7.5%.	44 456 (43)	62 median (IQR 52–72)	Prescription fill data for ibuprofen, naproxen or diclofenac from National Prescription Registry. New user design with time-varying analgesic exposure.	Primary composite: myocardial infarction, ischaemic stroke, congestive heart failure, atrial fibrillation or flutter, and all-cause death based on linked registry data.	Pooled logistic regression model weighted by age, sex, baseline rDANCAMI, and baseline drug use to calculate stabilised inverse probability of treatment weights for exposure to ibuprofen, naproxen or diclofenac.	*No consistent association between NSAID use and composite cardiovascular events stratified by MM measure*: ibuprofen: rDANCAMI score: 0=1.58 (1.35–1.86); score 1–3=1.39 (1.12–1.73); score 4–5=1.29 (0.82–2.04); score ≥6=1.33 (1.02–1.75). Naproxen: rDANCAMI score: 0=1.68 (0.83–3.38); score 1–3=1.48 (0.61–3.61); score 4–5=unable to compare (too few events); score ≥6=0.01 (0.00–0.10). Diclofenac: rDANCAMI score: 0=3.77 (2.49–5.71); score 1–3=2.09 (1.10–3.99); score 4–5=0.73 (0.19–2.78); score ≥6=0.91 (0.36–2.28).
Liu and colleagues, 2018^81^	190	JH ADG score measured within 5 yr before index date. Number of conditions used N/R. MM measure of sub-populations: TMA positive (ADG mean 14 [sd 3.43], median 14 [IQR 12–16]) *vs* TMA negative (ADG mean 12 [sd 3.77], median 12 [IQR 9–15]).	120 (63.2)	67 (16)	Drug dispensation data from administrative database. ≥1 NSAID or acetaminophen dispensed (dichotomised as NSAID [exposed] *vs* acetaminophen [unexposed]) during study dates. No dosing information. Prevalent user design.	TMA hospitalisation/diagnosis within 1.5 times the variable ‘day supply’ after prescription start date.	Unadjusted results *NB: cases matched to controls based on* age, sex, index date, rural residence, neighbourhood income quintile and conditions, and drugs associated with TMA (malignant HTN, SLE, HIV, sepsis, quetiapine, tacrolimus, sirolimus, cyclosporine, clopidogrel, and ticlopidine).	*NSAIDs associated with a relative reduction in occurrence of TMA among MM adults:* 19/38 cases (50%) had recent exposure to NSAIDs. 115/152 Controls (76%) had recent exposure to NSAIDs. Unadjusted OR: paracetamol=1.0 (ref). NSAIDs 0.32 (95% CI 0.15–0.69).
Yamanashi and colleagues, 2023^83^	1274	CCI measured at study enrolment. Number of conditions N/R. Mean (sd) CCI study population 3.3 (3). Mean (sd) CCI sub-populations, NSAID group 2.8 (2.9), no NSAID 3.6 (2.9).	619 (48.6)	68.8 (13.6)	Medication use history for NSAIDs by study enrolment based on EHR. No dosing information. Prevalent user design.	Delirium based on any questionnaire (i.e. DOSS/DRS-R-98/CAM-ICU) screening positive or clinical description in medical record showing the evidence of confusion or mental status change consistent with delirium. Assessed on admission/study enrolment.	Multivariate logistic regression adjusted for age, sex, dementia status, hospitalisation department, aspirin, (NSAIDs), glucosamine, and other anti-inflammatory drugs.	*NSAIDs not associated with delirium in adults with MM*: crude event rate for delirium: NSAID group: 101/439 (23%) *vs* no NSAID group: 292/835 (35%). NSAIDs OR 0.76 (95% CI 0.55–1.03) *P* 0.077. *Meloxicam associated with a reduction in delirium in adults with MM*: individual types of NSAID: in the analysis using all subjects (*n*=1274), meloxicam was significantly associated with a lower risk of delirium (OR: 0.46, 95% CI 0.22–0.97, *P*=0.04) while other individual NSAIDs were not.
Humes and colleagues, 2011^75^	9879	CCI measured 1 month before index. Number of conditions N/R. MM measure of sub-populations: cases CCI 0: 357 (39.71%). CCI 1: 215 (23.92%). CCI ≥2: 327 (36.37%) *vs* controls: CCI 0: 5133 (57.16%). CCI 1: 1867 (20.79%). CCI ≥2: 1980 (22.05%).	6065 (61.4)	N/R (category only).	Prescription fill data from UK GPRD database. Current users=prescription of a drug in the 6 months before index. Ever users=prescription at any time before this 6-month window without a current prescription. No dosing information. Prevalent user design.	Free diverticular perforation via GPRD coding anytime during study period (1990–2005).	Multivariate logistic regression adjusted for age and sex.	*NSAID use not associated with free diverticular perforation amongst adults with or without MM:* stratum specific OR (95% CI) by CCI for NSAID use NSAID current *vs* never: CCI 0: 1.48 (95% CI 0.81–2.71), CCI 1: 1.32 (95% CI 0.55–3.14), CCI ≥2: 1.90 (95% CI 0.89–4.06).
Zemedikun and colleagues, 2022^74^	661 499	Measured by LTC count any time before diagnosis of OA based on 30 diseases. LTC median (IQR) study sub-populations OA group LTC 2 (1–4) *vs* non-OA group LTC 1 (0–3)	396 802 (60.0)	62.8 (12.4)	Prescription data from IMRD-UK EHR database. No dosing information. Prevalent user design.	All-cause mortality anytime during follow-up post lag period of 1 yr from index date (maximum 9 yr).	Cox proportional hazard analysis with LCA matched 1:1 (based on age, sex, and general practice) adjusted for comorbidity phenotypes, age, sex, BMI categories, deprivation quintiles, smoking status, and ethnicity.	*NSAIDs associated with a reduction in mortality in adults with MM*: cases (i.e. MM): no opioid (reference); NSAIDs HR 0.95 (95% CI 0.93–0.98). *NSAIDs not associated with mortality in adults without MM:* controls (i.e. non-MM): no opioid (reference); NSAIDs HR 1.04 (95% CI 0.99–1.10).

#### Evidence of drug-related harm (n=6)

##### Secondary care settings (n=3)

Three studies using secondary care datasets reported analgesic-related harms (i.e. postoperative blood transfusion, treatment-resistant depression and AKI),^78^, ^79^, ^80^ two of which were based on hospital inpatients.^79^^,^^80^ Current NSAID use compared with non-use was associated with a modest increase in red cell transfusion within 7 days of hip fracture corrective surgery among older adults (≥65 yr) with multimorbidity.^79^ Non-selective NSAIDs, but not cyclooxygenase-2 (COX-2) inhibitors, were associated with an increased risk of treatment-resistant depression among outpatients with multimorbidity and major depression. However, analgesic exposure was based on a single prescription.^78^ Multimorbidity (i.e. LTC count) was associated with an increased risk of AKI at 7 days in older adults (≥60 yr) who received i.v. ketorolac for pain during hospital admission after traumatic injury. The absolute number of AKI episodes was low and many confounders were not considered.^80^

##### Primary and secondary care settings (n=3)

Three studies used primary and secondary care data to assess adverse outcomes (i.e. gastrointestinal [GI] bleeding, cardiovascular disease, and death after NSAID-associated event) among outpatient NSAID users.^76^^,^^77^^,^^82^ Multimorbidity (increasing Deyo comorbidity index score) was an independent predictor of death after an NSAID-associated event (i.e. upper GI endoscopy, myocardial infarction or stroke) among older veterans.^76^ Multimorbidity (increasing CCI) had a modest predictive effect on GI bleeding in adults treated with NSAIDs, which was more pronounced among a low-risk sub-population (i.e. age <65 yr, no recent warfarin or steroid treatment or recent GI bleed).^82^ Non-selective NSAIDs, COX-2 inhibitors, and simultaneous use of ≥2 NSAIDs were associated with an increased risk of composite cardiovascular disease compared with non-use among a multimorbid population of adults with rheumatoid arthritis.^77^

#### No evidence of drug-related harm (n=5)

Five studies reported no clear evidence of harm associated with NSAIDs among adults with multimorbidity including cardiovascular events, thrombotic microangiopathy (TMA), delirium, diverticular perforation, and mortality.^74^^,^^75^^,^^81^^,^^83^^,^^85^ A population-based study of adults with type 2 diabetes showed no graded increase in the association between NSAID use and composite cardiovascular events when stratified by multimorbidity measure (i.e. rDANCAMI score).^85^ Two studies used secondary care datasets to assess hospital inpatients.^81^^,^^83^ NSAIDs were associated with a relative reduction in the odds of hospitalisation for TMA compared with paracetamol. TMA was a rare event in the study population.^81^ NSAIDs were not associated with delirium amongst a population of older, multimorbid volunteers, however, in drug-specific analysis, meloxicam use was associated with a lower risk of delirium.^83^ Finally, two studies based in UK primary care compared current *vs* no NSAID use in sub-populations with multimorbidity.^74^^,^^75^ One showed no association between use and free diverticular perforation in the sub-population with multimorbidity.^75^ While the other reported NSAIDs were associated with a marginal reduction in mortality among a sub-population of adults with multimorbidity compared with no association among a sub-population of adults without multimorbidity.^74^

### Gabapentinoids

Characteristics of the single study reporting gabapentinoids are summarised in Table 5. Postoperative gabapentin use compared with non-use was associated with delirium and pneumonia, but not mortality in adults with multiple LTCs (i.e. combined comorbidity index ≥4).^84^

**Table 5:** Characteristics and results of studies of gabapentinoids. CCIn, combined comorbidity index; CI, confidence interval; CT, computed tomography; MM, multimorbidity; N/R, not reported; POD, postoperative day; PSM, propensity score matching; RD, risk difference; RR, relative risk; sd, standard deviation. ∗Crude numbers refer to those pre-PSM (i.e. propensity score matching). ^†^Age in yr presented as mean (sd).

Reference	Sample size	Patient characteristics			Analgesic exposure	Adverse outcome	Analysis	Main findings
		Measurement of multimorbidity	Female, *n* (%)	Age^†^				
Park and colleagues, 2022^84^	967 547^∗^	Combined comorbidity index. Timing and number of conditions: N/R. Compared MM *vs* non-MM (index ≥4 *vs* <4). Mean comorbidity index (sd) of sub-populations: gabapentin group 1.2 (2.2) and no gabapentin group 1.8 (2.6).	576 658 (59.6)	76.2 (7.4)	Charge codes (i.e. medications for billing and reimbursement) on POD 0, 1 or 2 from nationwide administrative database. Total daily gabapentin dose in milligrams given during the exposure defining period (i.e. POD 0, 1, and 2). New user design.	In-hospital death. Delirium including explicit (i.e. delirium is directly mentioned) and implicit (e.g. encephalopathy) criteria. Pneumonia via diagnostic code+i.v. antibiotic use or CT chest. All outcomes determined until hospital discharge.	Multivariable logistic regression models. Gabapentin *vs* no gabapentin via PSM which included patient characteristic information, insurance type, admission characteristics, surgery type, (combined comorbidity score), comorbidities, inpatient medication use, and procedures before or on POD 2, hospital-level characteristics, geographic region, and calendar year.	*Gabapentin has no association with in-hospital mortality in subgroups with or without MM*. Gabapentin *vs* non-gabapentin: CCI <4: RR 0.88 (95% CI 0.67–1.15); CCI ≥4: RR 1.05 (95% CI 0.88–1.24). *Gabapentin associated with delirium in subgroups with and without MM.* Gabapentin *vs* non-gabapentin CCI <4: RR 1.2 (95% CI 1.13–1.27). CCI ≥4: RR 1.4 (95% CI 1.30–1.51). RD 0.41 (95% CI 0.28–0.53) *vs* 2.66 (95% CI 2.08–3.24) per 100 persons; *P*<0.001 for heterogeneity. *Gabapentin associated with pneumonia in subgroups with and without MM.* Gabapentin *vs* non-gabapentin CCI <4: RR 1.22 (95% CI 1.10–1.35). CCI ≥4: RR 1.19 (95% CI 1.07–1.33). RD 0.15 [95% CI 0.07–0.22] *vs* 0.66 (0.25–1.07) per 100 persons; *P*=0.02 for heterogeneity.

## Discussion

This review identified 27 studies that reported harms from opioids, gabapentinoids or NSAIDs prescribed for pain in adults with multimorbidity, or the impact of multimorbidity on adverse outcomes among adults prescribed analgesics. Studies were heterogenous in their study design and of variable quality. Few reported absolute adverse event rates and data on analgesic dosing were often absent. Opioid use, compared with non-use, was associated with increased mortality among adults with multimorbidity. Multimorbidity, by different measures, was associated with opioid overdose, abuse, and mortality among adults prescribed opioids for pain. Half of studies of NSAIDs (six out of 11) reported drug-related harms across different outcome measures. Only one study reported the risks of gabapentinoids, which found an association with delirium and pneumonia, but not death, among adults with multiple LTCs.

Chronic pain leads to reduced quality of life,^87^^,^^88^ which itself is the highest ranked patient-reported outcome amongst adults with multimorbidity.^89^ Optimal pain management requires a comprehensive assessment to determine aetiology, timing (e.g. acute or chronic) and classification (e.g. primary or secondary). The complexity of chronic pain management is compounded by psychiatric, psychological, and medical comorbidities^90^ with associated polypharmacy. Cardiovascular, neurological, musculoskeletal, gastrointestinal, and mental health conditions predict higher levels of pain in adults with multimorbidity^10^^,^^11^^,^^17^ with corresponding implications for treatment. There is a clear biological rationale for altered analgesic pharmacokinetics among certain physical comorbidities (e.g. reduced renal clearance of opioid metabolites in chronic kidney disease),^91^ however, mechanisms are less well defined in psychiatric illness (e.g. responsiveness to opioid analgesics in depression/anxiety).^92^ Recent evidence has highlighted the importance of adverse childhood experiences (ACEs) on the development of multimorbidity,^93^ chronic pain, and major depression^94^ in adulthood. ACEs also increase the risk of prescription opioid misuse.^95^ Despite an understanding of the translational mechanisms of analgesic properties, there is limited evidence of the relationships among different disease and drug clusters to guide clinicians when managing pain in patients with multimorbidity.^96^

Opioid use has increased dramatically in recent decades.^97^^,^^98^ Data from UK primary care highlights a five-fold and 30-fold increase in codeine and oxycodone prescribing, respectively, for non-cancer pain from 2006 to 2017,^97^ with 18% of the Scottish population being prescribed an opioid in 2012.^99^ Multimorbidity increases the likelihood of persistent use after first prescription^100^, ^101^, ^102^, ^103^, ^104^ and people with multiple LTCs are over-represented among patients receiving strong opioids.^97^ One in five adults newly prescribed very high doses of opioids (i.e. >200 MME per day) remain on such doses for at least 2 years,^97^ emphasising the importance of understanding long-term drug-related harms, particularly as trials both under-report adverse events^105^ and are usually limited to short-term outcomes.^25^^,^^105^ Randomised controlled trials of opioids for chronic non-cancer pain exclude patients with past or present substance use disorder or active mental illness, despite the high prevalence of both, amongst patients prescribed opioids.^25^^,^^106^^,^^107^ Qualitative evidence among patients with multiple LTCs and chronic pain highlights concerns related to both opioid harms (e.g. dose titration, addiction, and overmedication) and potential undertreatment (e.g. barriers to access).^108^

This review is the first to our knowledge to synthesise evidence on the risks of analgesics in adults with multimorbidity. Multimorbidity is a relatively novel concept in health research which is reflected in the small number of studies included in our review. The absence of evidence, particularly from primary care settings, is an important finding. However, we adopted an extensive and systematic search of the literature and considered a large proportion of full-text reviews in an attempt to ensure adequate capture of multimorbidity and adverse events given under-reporting in titles/abstracts.^49^ In addition, this review included a broad range of potential drug-related harms and highlights clear limitations in the available evidence.

This review has limitations. The focus was to establish adverse analgesic-related outcomes without measuring benefits, however, syntheses of benefits and harms in reviews frequently under-represent the latter.^109^ Limitations in the synthesis reflect limitations in the primary literature (e.g. lack of adjustment for confounding by indication) as is common in many reviews of harms.^110^ The review included a broad range of studies from a variety of settings with differences in exposures, comparators, and outcomes. As such, statistical synthesis was not possible and narrative synthesis was challenging. This heterogeneity was thought to reflect the literature on multimorbidity and the breadth of the review question which aimed to synthesise knowledge for an underserved population using an explorative, hypothesis-generating approach. However, it is difficult to draw definitive conclusions from these findings and further work of sufficient granularity, in particular in regard to specific inclusion of individual comorbidities, is required. Some studies were included based on a population or sub-population mean who were multimorbid (as defined by a recommended measure of multimorbidity). Furthermore, multimorbidity was deduced from a CCI of greater than two for some studies despite CCI score weighting for condition severity.^111^ However, CCI is one of the most commonly used and validated measures of multimorbidity.^44^^,^^111^, ^112^, ^113^ Other definitions based on proxy measures of multimorbidity (e.g. RUB 3–5) were used. The findings may therefore contain adults with and without multimorbidity hence diluting reported relationships between multimorbidity and analgesic-related harms. However, our approach was adopted to be intentionally inclusive to draw conclusions in an area which is understudied. The review did not include paracetamol as it is usually purchased over the counter and we considered it to have limited harms when used at an appropriate dose.^114^ However, recent evidence suggestive of harms amongst older adults^115^ merits further study. Antidepressants were not included as the primary indication is for depressive symptoms rather than pain. Finally, results of studies which comprised mutual adjustment for both the relevant analgesic and multimorbidity measure (hence removing the ability to isolate the relationship of interest) could not be included.

Guidelines recommend that treatment strategies for chronic pain should be individualised and multimodal including exercise programmes, psychological therapy, and pharmacotherapy with multidisciplinary co-ordination.^14^^,^^116^ Our review highlights the potential for harms from drug treatments in people with multimorbidity, emphasising the need for a holistic, patient-centred approach incorporating physical and mental health rather than a disease-specific focus.^2^^,^^8^

Studies have reported adverse outcomes associated with polypharmacy in people with multimorbidity,^117^, ^118^, ^119^ however, our review highlights a lack of evidence relating to analgesic harms. This discrepancy may reflect the challenges of designing a robust pharmacoepidemiological study with an appropriate active comparator group to ensure confounding by indication is avoided. Randomised controlled trials are needed which actively recruit adults with multiple LTCs and report absolute event rates alongside benefits with a focus on health-related quality of life, mental health outcomes, and mortality.^89^ Routinely collected data offer an opportunity to understand adverse drug events in real-world settings using large populations to generate timely evidence for limited cost.^120^ Novel epidemiological techniques, such as target trial emulation,^121^ using real-world data may allow quantification of long-term analgesic outcomes while minimising the impact of common sources of bias (e.g. prevalent user) by adopting a causal inference approach. Studies are needed particularly in relation to harms associated with NSAIDs and gabapentinoids among people with multimorbidity given the lack of evidence. Future studies should include clear definition and measurement of multimorbidity and adopt approaches that are consistent with consensus opinion including reporting the prevalence of individual co-existent conditions.^46^ This would allow identification of specific patterns of LTCs (e.g. mental–physical multimorbidity) which may confer greater risk of adverse analgesic-related outcomes. Health policy must re-orientate to account for multimorbidity given that patients with multiple LTCs account for more than half of primary and secondary care costs.^122^

Pain is a common and debilitating symptom in patients with multimorbidity. Optimal management strategies are currently unknown, but despite these evidence gaps, analgesic use is widespread. Our review summarises the available evidence on the risks of analgesics in adults with multimorbidity suggesting the need for caution when prescribing opioids because of a potentially increased risk of overdose and mortality. There is a pressing need for research which addresses the benefits and harms of pain management, specifically NSAIDs and gabapentinoids, in adults with multiple LTCs. Such evidence would inform an individualised risk prediction approach accounting for complex disease–disease and disease–drug interactions, empowering both healthcare providers and people living with multimorbidity to make informed decisions about their healthcare.

## Authors’ contributions

Research idea and design: SB, CHG, PBM, LAC

Data acquisition: CHG, HW

Data interpretation: CHG, HW, KB, SB

Data synthesis: CHG, SB

Supervision: SB, PBM, LAC

Contributed important intellectual content during manuscript drafting or revision: all authors

Agree to be accountable for integrity of the work and will ensure that questions pertaining to the accuracy or integrity of any portion of the work, are appropriately resolved: CHG, SB

Approved the final manuscript: all authors

## Funding

CHG and HW are each funded separately by PhD scholarships at the University of Dundee & the University of Glasgow, respectively, as part of the Multimorbidity PhD Programme for Health Professionals supported by the Wellcome Trust (HW grant number: 223499/Z/22/Z). For the purpose of open access, the author has applied a CC BY public copyright licence to any Author Accepted Manuscript version arising from this submission.

## Declarations of interest

CHG, HW, and KB declare that they have no conflicts of interest. Outside the submitted work PBM has received honoraria for lectures and advisory boards from AstraZeneca, Vifor, Pharmacosmos, Astellas, GlaxoSmithKline (GSK), Bayer, and Boehringer Ingelheim and research funding from AstraZeneca and Boehringer Ingelheim. SB has received consultancy fees from AstraZeneca, GSK, and Bayer and research funding from AstraZeneca. LAC receives research support funding, on behalf of her institution, from the Advanced Pain Discovery Platform (funded by UK Research and Innovation, Versus Arthritis, Eli Lilly), the Scottish Government (Chief Scientist Office), The Wellcome Trust, and the National Institute of Academic Anaesthesia. She is Vice Chair of SIGN Council and is currently chairing the SIGN Guideline Development Group for Management of Chronic Pain.
