# Supplementary material for: Multimorbidity and analgesic-related harms: a systematic review
Source: Br J Anaesth. 2025 Mar 20;134(6):1717–45. doi: 10.1016/j.bja.2025.02.012 (PMC12106897; doi:10.1016/j.bja.2025.02.012)
Supplement: Multimedia component 1 [file mmc1.docx]

**Supplementary Information S1: Full details of inclusion & exclusion criteria**

***Population***

*Include*

- adults (aged 18 years or older)

*AND*

- studies reporting a recommended measure of multimorbidity (i.e. long-term condition count or weighted co-morbidity scoring system*)(1)

*AND*

- studies of multimorbid (i.e. two or more long-term conditions) populations, defined as any of:
  - a recommended measure of multimorbidity of >/=2 being a condition for study entry
  - a mean/median recommended measure of multimorbidity >/= 2 in the study population or analgesic-exposed sub-population
  - stratification/subgroup analyses of analgesic related adverse effects according to a recommended measure of multimorbidity

*OR*

- studies of general populations where the independent association of a recommended measure of multimorbidity on analgesic related adverse effects, is individually reported, in those exposed to relevant analgesics

*Exclude*

- children / young adults (aged under 18 years)
- studies which did not record or report a recommended measure of multimorbidity
- studies of general populations where the impact of a recommended measure of multimorbidity on analgesic related adverse effects was not individually reported in those exposed to relevant analgesics
- studies where the only extractable result of analgesic related adverse effects includes both the recommended multimorbidity measure and the relevant analgesic in the multivariable adjusted model (i.e. such that the relationship of interest cannot be determined)
- animal studies

***Intervention / Exposure***

*Include*

- NSAIDs (non-aspirin), opioids & gabapentinoids** for pain during the study period regardless of form, dose or duration of exposure.

*Exclude*

- Studies without exposure to NSAIDs, opioids &/or gabapentinoids
- Studies of listed analgesics for indications other than pain (e.g. substance use disorder, restless leg syndrome or induction anaesthesia)
- Studies where the isolated impact of an analgesic can’t be determined (e.g. combination drugs)

***Comparator / Context***

*Include*

- Studies that compare two or more different analgesic drugs
- Studies that compare analgesic vs no analgesic
- Studies that compare analgesic vs placebo
- Studies that compare multimorbid vs non-multimorbid subgroups
- Studies without a comparison group

*Exclude*

- Nil

***Outcome***

*Include*

- Adverse events related to analgesic use defined according to the PRISMA harms checklist as ‘an unfavourable outcome that occurs during or after the use of a drug’ for which ‘the causal relation between the intervention and the event is at least a reasonable possibility’.(2-4) Drug-related harms therefore included any harm reported regardless of whether it was considered serious, that could be potentially related to the consumption of a relevant analgesic^£^. We will not consider potentially inappropriate medications or drug interactions to represent adverse events in and of themselves unless, as above, such issues precipitate a defined unfavourable outcome.

*Exclude*

- Nil

***Study Characteristics***

*Include*

- Empirical quantitative studies^$^ i.e. observational (retrospective, prospective or cross-sectional) or interventional (randomised controlled trials, RCTs, or quasi-randomised controlled trials, qRCTs)
- English language

*Exclude*

- Qualitative studies, case reports, case series, letters, commentaries, book chapters, abstracts, editorials, review articles (including narrative, systematic, scoping & meta-analyses), conference proceedings, consensus statements, non-RCT or non-q-RCT interventional studies, trial protocols or economic evaluations
- Non-English language studies
- Full text unavailable

******* *We include the use of weighted co-morbidity scoring systems due to the predictive importance of individual measures (eg the Charlson Co-Morbidity Index and mortality) in addition to long term condition count(s) given that we are seeking to capture multiple outcomes for which such indices have not been not validated.(5-8) We will not include simple medication counts as a proxy of multimorbidity given that multiple medications can be used to treat a single condition (eg hypertension).*

*** A list of these drugs is based on the relevant sections of the British National Formulary(9) at the time of prospective systematic review registration on PROSPERO. In observational studies, exposure will be determined by any means including for example prescribing data, pharmacy claims, self-reported use or over the counter sales.*

*^£^ We are aiming to evaluate any or all possible harms in adults with multimorbidity including new or unexpected events and, as such, we will not pre-define adverse outcomes of interest nor duration of follow up in the included studies. This exploratory (ie hypothesis generating) approach will allow us to capture a broad overview of the safety of analgesics in adults with multimorbidity.(10)*

^$^ We expect that few randomised controlled trials (RCTs) will have been conducted in adults with measured multimorbidity. Observational studies will therefore be included to identify long term, rare or unexpected adverse outcomes as RCTs are usually limited to short term follow up.(10, 11)

**Supplementary Information S2a: Newcastle-Ottawa Quality Assessment Scale for Cohort studies**

**Selection (4*)**

1) Representativeness of the exposed cohort

a) truly representative of the average in the target population (ie adults with MM) [eg multi-centre & random sampling] *

b) somewhat representative of the average in the target population (ie adults with MM) [single centre or non-random sampling] *

c) selected group of users (eg specific sub-group of adults or other significant concern regarding selection bias)

d) no description of the derivation of the cohort

2) Selection of the non exposed cohort

a) drawn from the same community as the exposed cohort *

b) drawn from a different source

c) no description of the derivation of the non exposed cohort

d) no control group

3) Ascertainment of exposure

a) secure record (eg community prescription records) with new user design employed & time varying analgesic drug exposure established *

b) structured interview with new user design employed & time varying analgesic drug exposure established *

c) written self report or secure record / structured interview without new user design employed or time varying analgesic drug exposure established

d) no description

4) Demonstration that outcome of interest was not present at start of study

a) yes *

b) no

c) no description / unclear

**Comparability (2*)**

1) Comparability of cohorts on the basis of the design or analysis

a) study controls for concurrent use of other medications & any additional factor **

b) study controls for concurrent use of other medications (the most important factor) *

c) study controls for any additional factor(s) *

d) study does not control for any of the above factors

**Outcome (3*)**

1) Assessment of outcome

a) independent blind assessment or confirmation of the outcome by reference to secure primary records (ie medical records) *

b) record linkage (e.g. identified through ICD codes on database records) *

c) self report or other determination (ie no reference to original medical records to confirm the outcome)

d) no description

2) Was follow-up long enough for outcomes to occur

a) yes (ie at least 3 months) *

b) no

c) unclear / not reported

3) Adequacy of follow up of cohorts

a) complete follow up - all subjects accounted for *

b) subjects lost to follow up unlikely to introduce bias (ie < 20% loss to follow up) or adequate description provided of those lost *

c) follow up rate < 80% & no description of those lost

d) no statement

**Supplementary Information S2b: Newcastle-Ottawa Quality Assessment Scale for Case-control studies**

**Selection (4*)**

1) Is the case definition adequate?

a) yes, with independent validation (eg by reference to secure primary records ie medical records) *

b) yes, eg record linkage identified through ICD codes on database records *

c) no, eg self-reported

d) no description

2) Representativeness of the cases

a) consecutive or obviously representative series of cases *

b) potential for selection biases

c) not stated

3) Selection of Controls

a) community controls *

b) hospital controls

c) no description

4) Definition of Controls

a) no history of disease (endpoint) *

b) no description

**Comparability (2*)**

1) Comparability of cases and controls on the basis of the design or analysis

a) study controls for concurrent use of other medications & any additional factor **

b) study controls for concurrent use of other medications (the most important factor) *

c) study controls for any additional factor(s) *

d) study does not control for either of the above

**Exposure (3*)**

1) Ascertainment of exposure

a) secure record (eg community prescription records) with new user design & time varying analgesic drug exposure established *

b) structured interview where blind to case/control status with new user design & time varying analgesic drug exposure established *

c) interview not blinded to case/control status or without new user design employed or time varying analgesic drug exposure established

d) written self report or medical record only

e) no description

2) Same method of ascertainment for cases and controls

a) yes *

b) no

3) Non-Response rate

a) same rate for both groups *

b) non respondents described

c) rate different

d) no description provided

**Supplementary Information S2c: Newcastle-Ottawa Quality Assessment Scale for Cross-sectional studies**

**Selection (4*)**

1) Representativeness of exposed subjects

a) truly representative of the average in the target population (ie adults with MM) [eg multi-centre & random sampling] *

b) somewhat representative of the average in the target population (ie adults with MM) [single centre or non-random sampling] *

c) selected group of users (eg specific sub-group of adults or other significant concern regarding selection bias)

d) no description of the derivation of the cohort

2) Sample size

a) justified & satisfactory *

b) less than 30 participants assessed for outcome

c) no information provided

3) Non-respondents

a) Proportion of the target sample recruited is satisfactory (ie >80%) or a comparative summary of non-respondents is provided *

b) Proportion of the target sample recruited is unsatisfactory (ie < 80%) & no summary data provided on non-respondents.

c) No information provided

4) Ascertainment of exposure

a) secure record (eg community prescription records) with attempt to quantify ‘new use’ (ie < 3 months) of relevant analgesic *

b) structured interview with attempt to quantify ‘new use’ (ie < 3 months) of relevant analgesic *

c) written self report or only prevalent use quantified

d) no description

**Comparability (2*)**

1) Comparability of cohorts on the basis of the design or analysis

a) study controls for both concurrent use of other medications & any additional factors (the most important factor) **

b) study controls for concurrent use of other medications *

c) study controls for any additional factor(s) *

d) study does not control for either of the above

**Outcome (3*)**

1) Assessment of outcome

a) independent blind assessment or confirmation of the outcome by reference to secure primary records (ie medical records) **

b) record linkage (e.g. identified through ICD codes on database records) **

c) self report or other determination (e.g. no reference to original medical records to confirm the outcome)

d) no description

2) Statistical tests

a) The statistical test used to analyze the association is clearly described and appropriate, and the measurement is presented as either an OR, CI and P value or a beta coefficient, SE and P value *

b) The statistical test is inappropriate, poorly described or incomplete

c) No information provided

**Supplementary Information S3: Risk of bias assessment for the interventional study**

See separate document

**Supplementary Table S1a: Medline (via PubMed) search strategy**

|  | analgesic* OR opiate* OR opioid* OR Narcotic* OR Morphine OR Oxycodone OR Methadone OR Hydromorphone OR Hydrocodone OR Fentanyl OR Buprenorphine OR Tramadol OR Alfentanil OR Codeine OR Dihydrocodeine OR Remifentanil OR Sufentanil OR Meperidine OR Pethidine AND adverse effect* OR adverse reaction* OR adverse event* OR "opiate alkaloids/adverse effects"[MeSH Terms] "Narcotics/adverse effects"[MeSH Terms] OR "Methadone/adverse effects"[MeSH Terms] OR "Fentanyl/adverse effects"[MeSH Terms] OR "Tramadol/adverse effects"[MeSH Terms] OR "Meperidine/adverse effects"[MeSH Terms] OR  "analgesics/adverse effects"[MeSH Terms] OR "analgesics, opioid"[MeSH Terms] OR "nsaid*" OR "non steroidal anti inflammatory" OR "nonsteroidal anti inflammatory") AND "adverse effect*" OR adverse reaction* OR adverse event* OR "anti inflammatory agents, non steroidal/adverse effects"[MeSH Terms] OR "ibuprofen/adverse effects"[MeSH Terms] OR "naproxen/adverse effects"[MeSH Terms] OR "sulindac/adverse effects"[MeSH Terms] OR "ketoprofen/adverse effects"[MeSH Terms] OR "flurbiprofen/adverse effects"[MeSH Terms] OR "fenoprofen/adverse effects"[MeSH Terms] OR "etodolac/adverse effects"[MeSH Terms] OR "clonixin/adverse effects"[MeSH Terms] OR "diflunisal/adverse effects"[MeSH Terms] OR "epirizole/adverse effects"[MeSH Terms] OR "feprazone/adverse effects"[MeSH Terms] OR "ketorolac/adverse effects"[MeSH Terms] OR "meloxicam/adverse effects"[MeSH Terms] OR "diclofenac/adverse effects"[MeSH Terms] OR "mefenamic acid/adverse effects"[MeSH Terms] OR "meclofenamic acid/adverse effects"[MeSH Terms] OR "tolmetin/adverse effects"[MeSH Terms] OR "niflumic acid/adverse effects"[MeSH Terms] OR "phenylbutazone/adverse effects"[MeSH Terms] OR "piroxicam/adverse effects"[MeSH Terms] OR "etoricoxib/adverse effects"[MeSH Terms] OR "indomethacin/adverse effects"[MeSH Terms] OR "cyclooxygenase 2 inhibitors/adverse effects"[MeSH Terms] OR "bufexamac/adverse effects"[MeSH Terms] OR "antipyrine/adverse effects"[MeSH Terms] OR "nabumetone/adverse effects"[MeSH Terms] OR "suprofen/adverse effects"[MeSH Terms] OR "celecoxib/adverse effects"[MeSH Terms] OR (("Ibuprofen" OR "Naproxen" OR "Sulindac" OR "Ketoprofen" OR "Flurbiprofen" OR "Fenoprofen" OR "Etodolac" OR "Clonixin" OR "Diflunisal" OR "Tenoxicam" OR "Tenoxicam"[Supplementary Concept] OR "Epirizole" OR "Feprazone" OR "Ketorolac" OR "Meloxicam" OR "Diclofenac" OR "mefenamic acid" OR "meclofenamic acid" OR "Tolmetin" OR "niflumic acid" OR "Phenylbutazone" OR "Piroxicam" OR "Etoricoxib" OR "Valdecoxib" OR "Valdecoxib"[Supplementary Concept] OR "Rofecoxib" OR "Rofecoxib"[Supplementary Concept] OR "Indomethacin" OR "Apazone" OR "cox 2 inhibitor*" OR "coxib*" OR "cyclooxygenase 2 inhibitor*" OR "Bufexamac" OR "Fenbufen" OR "Floctafenine" OR "Floctafenine"[Supplementary Concept] OR "Fenbufen" OR "Fenbufen"[Supplementary Concept] OR "Phenazone" OR "Acemetacin" OR "Acemetacin"[Supplementary Concept] OR "Aceclofenac" OR "Aceclofenac"[Supplementary Concept] OR "Clofenamic" AND "acid") OR "Dexketoprofen" OR "dexketoprofen trometamol"[Supplementary Concept] OR "Dexibuprofen" OR "Dexibuprofen"[Supplementary Concept] OR "Lornoxicam" OR "Lornoxicam"[Supplementary Concept] OR "Nabumetone" OR "Nimesulide" OR "Nimesulide"[Supplementary Concept] OR "Suprofen" OR "tiaprofenic acid" OR "tiaprofenic acid"[Supplementary Concept] OR "Celecoxib" OR "Parecoxib" OR "Parecoxib"[Supplementary Concept] OR "Nurofen" AND "adverse effect*" OR adverse reaction* OR adverse event* OR "gabapentinoid*" OR "Gabapentin" OR "Pregabalin" AND ("adverse effect*" OR adverse reaction* OR adverse event* OR "gabapentin/adverse effects"[MeSH Terms] OR "pregabalin/adverse effects"[MeSH Terms] |
| --- | --- |
|  | "multimorbidity"[MeSH Terms] OR "multiple chronic conditions"[MeSH Terms] OR "multiple conditions" OR "multimorbid*" OR "multi morbid*" OR Charlson OR "multiple comorbidities" OR "multiple co morbidities" OR "discordant comorbidities" OR "concordant comorbidities" OR "comorbidity"[MeSH Terms] OR "comorbid*" OR "co morbid*" |
|  | "Risk Factors"[Mesh] OR Risk*[Title/Abstract] OR Adverse[Title/Abstract] OR Negative[Title/Abstract] |
|  | 1 and 2 and 3 |

**Supplementary Table S1b: CINAHL (Ebsco Host) & Web of Science search strategies**

| "analgesic*" OR "opiate*" OR "opioid*" OR Narcotic* OR Morphine OR Oxycodone OR Methadone OR Hydromorphone OR Hydrocodone OR Fentanyl OR Buprenorphine OR Tramadol OR Alfentanil OR Codeine OR Dihydrocodeine OR Remifentanil OR Sufentanil OR Meperidine OR Pethidine OR "opiate alkaloids" "Narcotics " OR "Methadone" OR "Fentanyl" OR "Tramadol" OR "Meperidine" OR  "analgesics" OR "analgesics, opioid" OR "nsaid*" OR "non steroidal anti inflammatory" OR "nonsteroidal anti inflammatory" OR "anti inflammatory agents, non steroidal" OR "ibuprofen" OR "naproxen" OR "sulindac" OR "ketoprofen" OR "flurbiprofen" OR "fenoprofen" OR "etodolac" OR "clonixin" OR "diflunisal" OR "epirizole" OR "feprazone" OR "ketorolac" OR "meloxicam" OR "diclofenac" OR "mefenamic acid" OR "meclofenamic acid" OR "tolmetin" OR "niflumic acid" OR "phenylbutazone" OR "piroxicam" OR "etoricoxib" OR "indomethacin" OR "cyclooxygenase 2 inhibitors" OR "bufexamac" OR "antipyrine" OR "nabumetone" OR "suprofen" OR "celecoxib" OR "Ibuprofen" OR "Naproxen" OR "Sulindac" OR "Ketoprofen" OR "Flurbiprofen" OR "Fenoprofen" OR "Etodolac" OR "Clonixin" OR "Diflunisal" OR "Tenoxicam" OR "Tenoxicam" OR "Epirizole" OR "Feprazone" OR "Ketorolac" OR "Meloxicam" OR "Diclofenac" OR "mefenamic acid" OR "meclofenamic acid" OR "Tolmetin" OR "niflumic acid" OR "Phenylbutazone" OR "Piroxicam" OR "Etoricoxib" OR "Valdecoxib" OR "Valdecoxib" OR "Rofecoxib" OR "Rofecoxib" OR "Indomethacin" OR "Apazone" OR "cox 2 inhibitor*" OR "coxib*" OR "cyclooxygenase 2 inhibitor*" OR "Bufexamac" OR "Fenbufen" OR "Floctafenine" OR "Phenazone" OR "Acemetacin" OR "Aceclofenac" OR "Clofenamic" OR "Dexketoprofen" OR "dexketoprofen trometamol" OR "Dexibuprofen" OR "Lornoxicam" OR "Nabumetone" OR "Nimesulide" OR "Suprofen" OR "tiaprofenic acid" OR "Celecoxib" OR "Parecoxib" OR "Nurofen" OR "gabapentinoid*" OR "Gabapentin" OR "Pregabalin" |
| --- |
| AND Multimorbidit* OR multi-morbidit* OR comorbidit* OR co-morbidit* OR Charlson OR “multiple conditions” OR “multiple chronic conditions” OR "multiple comorbidities" OR "multiple co morbidities" OR "discordant comorbidities" OR "concordant comorbidities" |
| AND adverse effect* OR adverse reaction* OR adverse event* OR adverse outcome* |

**Supplementary Table S1c: EMBASE (Ovid interface) search strategy**

| 1. ("analgesic*" or "opiate*" or "opioid*" or Narcotic* or Morphine or Oxycodone or Methadone or Hydromorphone or Hydrocodone or Fentanyl or Buprenorphine or Tramadol or Alfentanil or Codeine or Dihydrocodeine or Remifentanil or Sufentanil or Meperidine or Pethidine).mp. and (adverse effect* or adverse reaction* or adverse event* or adverse outcome*).ti,ab.  2. opiate/ae [Adverse Drug Reaction]  3. narcotic agent/ae [Adverse Drug Reaction]  4. methadone/ae [Adverse Drug Reaction]  5. fentanyl/ae [Adverse Drug Reaction]  6. tramadol/ae [Adverse Drug Reaction]  7. analgesic agent/ae [Adverse Drug Reaction]  8. nonsteroid antiinflammatory agent/ae [Adverse Drug Reaction]  9. ibuprofen/ae [Adverse Drug Reaction]  10. naproxen/ae [Adverse Drug Reaction]  11. sulindac/ae [Adverse Drug Reaction]  12. ketoprofen/ae [Adverse Drug Reaction]  13. flurbiprofen/ae [Adverse Drug Reaction]  14. fenoprofen/ae [Adverse Drug Reaction]  15. etodolac/ae [Adverse Drug Reaction]  16. clonixin/ae [Adverse Drug Reaction]  17. diflunisal/ae [Adverse Drug Reaction]  18. epirizole/ae [Adverse Drug Reaction]  19. feprazone/ae [Adverse Drug Reaction]  20. ketorolac/ae [Adverse Drug Reaction]  21. meloxicam/ae [Adverse Drug Reaction]  22. diclofenac/ae [Adverse Drug Reaction]  23. mefenamic acid/ae [Adverse Drug Reaction]  24. meclofenamic acid/ae [Adverse Drug Reaction]  25. tolmetin/ae [Adverse Drug Reaction]  26. niflumic acid/ae [Adverse Drug Reaction]  27. phenylbutazone/ae [Adverse Drug Reaction]  28. piroxicam/ae [Adverse Drug Reaction]  29. etoricoxib/ae [Adverse Drug Reaction]  30. indometacin/ae [Adverse Drug Reaction]  31. cyclooxygenase 2 inhibitor/ae [Adverse Drug Reaction]  32. bufexamac/ae [Adverse Drug Reaction]  33. phenazone/ae [Adverse Drug Reaction]  34. nabumetone/ae [Adverse Drug Reaction]  35. suprofen/ae [Adverse Drug Reaction]  36. celecoxib/ae [Adverse Drug Reaction]  37. 2 or 3 or 4 or 5 or 6 or 7 or 8 or 9 or 10 or 11 or 12 or 13 or 14 or 15 or 16 or 17 or 18 or 19 or 20 or 21 or 22 or 23 or 24 or 25 or 26 or 27 or 28 or 29 or 30 or 31 or 32 or 33 or 34 or 35 or 36  38. ("Ibuprofen" or "Naproxen" or "Sulindac" or "Ketoprofen" or "Flurbiprofen" or "Fenoprofen" or "Etodolac" or "Clonixin" or "Diflunisal" or "Tenoxicam" or "Tenoxicam" or "Epirizole" or "Feprazone" or "Ketorolac" or "Meloxicam" or "Diclofenac" or "mefenamic acid" or "meclofenamic acid" or "Tolmetin" or "niflumic acid" or "Phenylbutazone" or "Piroxicam" or "Etoricoxib" or "Valdecoxib" or "Valdecoxib" or "Rofecoxib" or "Rofecoxib" or "Indomethacin" or "Apazone" or "cox 2 inhibitor*" or "coxib*" or "cyclooxygenase 2 inhibitor*" or "Bufexamac" or "Fenbufen" or "Floctafenine" or "Fenbufen" or "Phenazone" or "Acemetacin" or "Aceclofenac" or "Clofenamic" or "Dexketoprofen" or "dexketoprofen trometamol" or "Dexibuprofen" or "Lornoxicam" or "Nabumetone" or "Nimesulide" or "Suprofen" or "tiaprofenic acid" or "Celecoxib" or "Parecoxib" or "Nurofen" or "gabapentinoid" or "antipyrine" or "meperidine").mp. and (adverse effect* or adverse reaction* or adverse event*).ti,ab.  39. gabapentin/ae [Adverse Drug Reaction]  40. pregabalin/ae [Adverse Drug Reaction]  41. 1 or 37 or 38 or 39 or 40  42. multiple chronic conditions/  43. ((Multimorbidit* or multi-morbidit* or comorbidit* or co-morbidit* or Charlson or "multiple conditions" or "multiple chronic conditions" or "multiple comorbidities" or "multiple co morbidities" or "discordant comorbidities" or "concordant comorbidities") and (adverse effect* or adverse reaction* or adverse event* or adverse outcome*)).ti,ab.  44. 42 or 43  45. risk factor/  46. (Risk* or Adverse or Negative).ti,ab.  47. 45 or 46  48. 41 and 44 and 47 |
| --- |

**Supplementary Table S1d: CENTRAL (Cochrane Library) search strategy**

| ID Search  #1 MeSH descriptor: [Opiate Alkaloids] explode all trees and with qualifier(s): [adverse effects - AE]  #2 MeSH descriptor: [Narcotics] explode all trees and with qualifier(s): [adverse effects - AE]  #3 MeSH descriptor: [Methadone] explode all trees and with qualifier(s): [adverse effects - AE]  #4 MeSH descriptor: [Fentanyl] explode all trees and with qualifier(s): [adverse effects - AE]  #5 MeSH descriptor: [Tramadol] explode all trees and with qualifier(s): [adverse effects - AE]  #6 MeSH descriptor: [Meperidine] explode all trees and with qualifier(s): [adverse effects - AE]  #7 MeSH descriptor: [Analgesics] explode all trees and with qualifier(s): [adverse effects - AE]  #8 MeSH descriptor: [Analgesics, Opioid] explode all trees and with qualifier(s): [adverse effects - AE]  #9 MeSH descriptor: [Anti-Inflammatory Agents, Non-Steroidal] explode all trees and with qualifier(s): [adverse effects - AE]  #10 MeSH descriptor: [Ibuprofen] explode all trees and with qualifier(s): [adverse effects - AE]  #11 MeSH descriptor: [Naproxen] explode all trees and with qualifier(s): [adverse effects - AE]  #12 MeSH descriptor: [Sulindac] explode all trees and with qualifier(s): [adverse effects - AE]  #13 MeSH descriptor: [Ketoprofen] explode all trees and with qualifier(s): [adverse effects - AE]  #14 MeSH descriptor: [Flurbiprofen] explode all trees and with qualifier(s): [adverse effects - AE]  #15 MeSH descriptor: [Fenoprofen] explode all trees and with qualifier(s): [adverse effects - AE]  #16 MeSH descriptor: [Etodolac] explode all trees and with qualifier(s): [adverse effects - AE]  #17 MeSH descriptor: [Clonixin] explode all trees and with qualifier(s): [adverse effects - AE]  #18 MeSH descriptor: [Diflunisal] explode all trees and with qualifier(s): [adverse effects - AE]  #19 MeSH descriptor: [Epirizole] explode all trees and with qualifier(s): [adverse effects - AE]  #20 MeSH descriptor: [Feprazone] explode all trees and with qualifier(s): [adverse effects - AE]  #21 MeSH descriptor: [Ketorolac] explode all trees and with qualifier(s): [adverse effects - AE]  #22 MeSH descriptor: [Meloxicam] explode all trees and with qualifier(s): [adverse effects - AE]  #23 MeSH descriptor: [Diclofenac] explode all trees and with qualifier(s): [adverse effects - AE]  #24 MeSH descriptor: [Mefenamic Acid] explode all trees and with qualifier(s): [adverse effects - AE]  #25 MeSH descriptor: [Meclofenamic Acid] explode all trees and with qualifier(s): [adverse effects - AE]  #26 MeSH descriptor: [Tolmetin] explode all trees and with qualifier(s): [adverse effects - AE]  #27 MeSH descriptor: [Niflumic Acid] explode all trees and with qualifier(s): [adverse effects - AE]  #28 MeSH descriptor: [Phenylbutazone] explode all trees and with qualifier(s): [adverse effects - AE]  #29 MeSH descriptor: [Piroxicam] explode all trees and with qualifier(s): [adverse effects - AE]  #30 MeSH descriptor: [Etoricoxib] explode all trees and with qualifier(s): [adverse effects - AE]  #31 MeSH descriptor: [Indomethacin] explode all trees and with qualifier(s): [adverse effects - AE]  #32 MeSH descriptor: [Cyclooxygenase 2 Inhibitors] explode all trees and with qualifier(s): [adverse effects - AE]  #33 MeSH descriptor: [Bufexamac] explode all trees and with qualifier(s): [adverse effects - AE]  #34 MeSH descriptor: [Antipyrine] explode all trees and with qualifier(s): [adverse effects - AE]  #35 MeSH descriptor: [Nabumetone] explode all trees and with qualifier(s): [adverse effects - AE]  #36 MeSH descriptor: [Suprofen] explode all trees and with qualifier(s): [adverse effects - AE]  #37 MeSH descriptor: [Celecoxib] explode all trees and with qualifier(s): [adverse effects - AE]  #38 MeSH descriptor: [Gabapentin] explode all trees and with qualifier(s): [adverse effects - AE]  #39 MeSH descriptor: [Pregabalin] explode all trees and with qualifier(s): [adverse effects - AE]  #40 (analgesic OR opiate OR opioid OR Narcotic OR Morphine OR Oxycodone OR Methadone OR Hydromorphone OR Hydrocodone OR Fentanyl OR Buprenorphine OR Tramadol OR Alfentanil OR Codeine OR Dihydrocodeine OR Remifentanil OR Sufentanil OR Meperidine OR Pethidine OR nsaid OR "non steroidal anti inflammatory" OR "nonsteroidal anti inflammatory" OR Ibuprofen OR Naproxen OR Sulindac OR Ketoprofen OR Flurbiprofen OR Fenoprofen OR Etodolac OR Clonixin OR Diflunisal OR Tenoxicam OR Epirizole OR Feprazone OR Ketorolac OR Meloxicam OR Diclofenac OR "mefenamic acid" OR "meclofenamic acid" OR "Tolmetin" OR "niflumic acid" OR "Phenylbutazone" OR "Piroxicam" OR "Etoricoxib" OR Valdecoxib OR Indomethacin OR "Apazone" OR "cox 2 inhibitor" OR "coxib" OR "cyclooxygenase 2 inhibitor" OR "Bufexamac" OR "Fenbufen" OR "Floctafenine" OR Phenazone OR Acemetacin OR Aceclofenac OR Clofenamic OR Dexketoprofen OR Dexibuprofen OR Lornoxicam OR Nabumetone OR Nimesulide OR Suprofen OR "tiaprofenic acid" OR Celecoxib OR Parecoxib OR Nurofen OR gabapentinoid* OR Gabapentin OR Pregabalin):ab  #41 "adverse effect" OR "adverse reaction" OR "adverse event" OR "adverse outcome"  #42 #40 and #41  #43 #1 or #2 or #3 or #4 or #5 or #6 or #7 or #8 or #9 or #10 or #11 or #12 or #13 or #14 or #15 or #16 or #17 or #18 or #19 or #20 or #21 or #22 or #23 or #24 or #25 or #26 or #27 or #28 or #29 or #30 or #31 or #32 or #33 or #34 or #35 or #36 or #37 or #38 or #39  #44 #42 or #43  #45 MeSH descriptor: [Risk Factors] explode all trees  #46 (risk OR negative OR adverse):ti  #47 (risk OR negative OR adverse):ab  #48 #45 or #46 or #47  #49 #44 and #48  #50 MeSH descriptor: [Multimorbidity] explode all trees  #51 MeSH descriptor: [Multiple Chronic Conditions] explode all trees  #52 MeSH descriptor: [Comorbidity] explode all trees  #53 ("multiple conditions" OR multimorbidities OR multimorbidity OR multi-morbidities OR multi-morbidity OR Charlson OR "multiple comorbidities" OR "multiple co-morbidities" OR "discordant comorbidities" OR "concordant comorbidities" OR comorbidity OR comorbidities OR co-morbidity OR co-morbidities):ti  #54 ("multiple conditions" OR multimorbidities OR multimorbidity OR multi-morbidities OR multi-morbidity OR Charlson OR "multiple comorbidities" OR "multiple co-morbidities" OR "discordant comorbidities" OR "concordant comorbidities" OR comorbidity OR comorbidities OR co-morbidity OR co-morbidities):ab  #55 #50 or #51 or #52 or #53 or #54  #56 #49 and #55 |
| --- |


**References**

1. Ho ISS, Azcoaga-Lorenzo A, Akbari A, Davies J, Khunti K, Kadam UT, et al. Measuring multimorbidity in research: Delphi consensus study. BMJ Medicine. 2022;1(1):e000247.

2. Zorzela L, Loke YK, Ioannidis JP, Golder S, Santaguida P, Altman DG, et al. PRISMA harms checklist: improving harms reporting in systematic reviews. BMJ. 2016;352:i157.

3. Chou R, Aronson N, Atkins D, Ismaila AS, Santaguida P, Smith DH, et al. AHRQ series paper 4: assessing harms when comparing medical interventions: AHRQ and the effective health-care program. Journal of clinical epidemiology. 2010;63(5):502-12.

4. Peryer G., Golder S., Junqueira D.R., Vohra S., Loke Y. K. Chapter 19: Adverse effects. In: Higgins JPT, Thomas J, Chandler J, Cumpston M, Li T, Page MJ, Welch VA (editors). Cochrane Handbook for Systematic Reviews of Interventions version 6.4 (updated August 2023). Cochrane, 2023. Available from <https://training.cochrane.org/handbook/current/chapter-19> [Accessed on 1^st^ September 2023]

5. Johnston MC, Crilly M, Black C, Prescott GJ, Mercer SW. Defining and measuring multimorbidity: a systematic review of systematic reviews. Eur J Public Health. 2019;29(1):182-9.

6. Huntley AL, Johnson R, Purdy S, Valderas JM, Salisbury C. Measures of multimorbidity and morbidity burden for use in primary care and community settings: a systematic review and guide. Ann Fam Med. 2012;10(2):134-41.

7. Vela E, Clèries M, Monterde D, Carot-Sans G, Coca M, Valero-Bover D, et al. Performance of quantitative measures of multimorbidity: a population-based retrospective analysis. BMC Public Health. 2021;21(1):1881.

8. Ho IS, Azcoaga-Lorenzo A, Akbari A, Black C, Davies J, Hodgins P, et al. Examining variation in the measurement of multimorbidity in research: a systematic review of 566 studies. Lancet Public Health. 2021;6(8):e587-e97.

9. Joint Formulary Committee. British National Formulary (online) London: BMJ and Pharmaceutical Press. Available from: <http://www.medicinescomplete.com>. [Accessed 4^th^ August 2023].

10. Loke YK, Golder SP, Vandenbroucke JP. Comprehensive evaluations of the adverse effects of drugs: importance of appropriate study selection and data sources. Ther Adv Drug Saf. 2011;2(2):59-68.

11. McKenzie JE, Brennan SE. Chapter 12: Synthesizing and presenting findings using other methods. In: Higgins JPT, Thomas J, Chandler J, Cumpston M, Li T, Page MJ, Welch VA (editors). Cochrane Handbook for Systematic Reviews of Interventions version 6.4 (updated August 2023). Cochrane, 2023. Available from <https://training.cochrane.org/handbook/current/chapter-12> [Accessed on 1^st^ September 2023]
